# Genetic variants in two pathways influence serum urate levels and gout risk: a systematic pathway analysis

**DOI:** 10.1038/s41598-018-21858-0

**Published:** 2018-03-01

**Authors:** Zheng Dong, Jingru Zhou, Xia Xu, Shuai Jiang, Yuan Li, Dongbao Zhao, Chengde Yang, Yanyun Ma, Yi Wang, Hongjun He, Hengdong Ji, Juan Zhang, Ziyu Yuan, Yajun Yang, Xiaofeng Wang, Yafei Pang, Li Jin, Hejian Zou, Jiucun Wang

**Affiliations:** 1 State Key Laboratory of Genetic Engineering, Collaborative Innovation Center for Genetics and Development, School of Life Sciences, Fudan University Jiangwan Campus, Shanghai, China; 2 Division of Rheumatology and Immunology, Changhai Hospital, Shanghai, China; 3 Division of Rheumatology, Ruijin Hospital, Shanghai Jiaotong University School of Medicine, Shanghai, China; 4 Ministry of Education Key Laboratory of Contemporary Anthropology, School of Life Sciences, Fudan University, Shanghai, China; 5 Division of Rheumatology, Taixing People’s Hospital, Jiangsu Province, China; 6 Division of Rheumatology, Taizhou People’s Hospital, Jiangsu Province, China; 7 Fudan-Taizhou Institute of Health Sciences, Taizhou, Jiangsu Province China; 8 Division of Rheumatology, Huashan Hospital, Fudan University, Shanghai, China; 9 Institute of Rheumatology, Immunology and Allergy, Fudan University, Shanghai, China

## Abstract

The aims of this study were to identify candidate pathways associated with serum urate and to explore the genetic effect of those pathways on the risk of gout. Pathway analysis of the loci identified in genome-wide association studies (GWASs) showed that the ion transmembrane transporter activity pathway (GO: 0015075) and the secondary active transmembrane transporter activity pathway (GO: 0015291) were both associated with serum urate concentrations, with *P*_FDR_ values of 0.004 and 0.007, respectively. In a Chinese population of 4,332 individuals, the two pathways were also found to be associated with serum urate (*P*_FDR_ = 1.88E-05 and 3.44E-04, separately). In addition, these two pathways were further associated with the pathogenesis of gout (*P*_FDR_ = 1.08E-08 and 2.66E-03, respectively) in the Chinese population and a novel gout-associated gene, *SLC17A2*, was identified (OR = 0.83, *P*_FDR_ = 0.017). The mRNA expression of candidate genes also showed significant differences among different groups at pathway level. The present study identified two transmembrane transporter activity pathways (GO: 0015075 and GO: 0015291) were associations with serum urate concentrations and the risk of gout. *SLC17A2* was identified as a novel gene that influenced the risk of gout.

## Introduction

Uric acid is the final catabolic product of purine oxidation in humans. Because of the loss of important genes in the urate degradation pathway during human evolution, uric acid cannot be metabolized into allantoin^1,2^. It is primarily produced in the liver, excreted via the kidney and the gut, and present in the blood as urate. Elevated serum urate (hyperuricemia) is caused by an imbalance between uric acid production and disposal^3^. This imbalance results in the deposition of monosodium urate (MSU) crystals in the joints leading to gout. Gout is one of the forms of common inflammatory arthritis, and it affects nearly 4% of American adults^4^. Recent epidemiological studies have suggested that the prevalence and incidence of gout are increasing^4–6^. In addition, gout is often associated with other diseases, such as hypertension, obesity, renal insufficiency, and heart failure^7^.

Serum urate concentrations exhibit a strong genetic predisposition, with a heritability estimate ranging from 40% to 70%^8^. Genome-wide association studies (GWASs) have identified several genetic variant loci in various genes associated with serum urate concentrations^8–15^. However, those genetic variants only explain 7.0% of the variance in serum urate concentrations^8^, which is distinctly less than what is estimated to be heritable. The key challenges for these GWASs are identifying causal SNPs and providing abundant evidence for the influence of candidate loci on serum urate concentrations by specific biological mechanisms^16^. Moreover, multiple genes may interact with each other and may work together to influence the development of a specific disease, especially in the case of complex disorders. In addition, pathway analysis has been shown to be a useful approach for clarifying biological insights and identifying new candidate genes^8^. Therefore, it is necessary to identify additional genetic factors influencing serum urate concentrations and the pathogenesis of gout using pathway analysis.

In the present study, we aimed to identify serum urate- and gout-associated candidate genes and biological pathways using a four-step approach. First, we applied pathway analysis to the SNPs identified as associated with serum urate concentrations in previous GWASs to identify candidate pathways and candidate genes in pathways. Then, the candidate genes and pathways were tested in 4,332 Chinese individuals to validate the associations between candidate genes and pathways and serum urate concentrations. Next, those candidate genes and pathways were also tested in a case-control study to find the candidate genes and pathways affecting the risk of gout. Finally, the transcription levels of candidate genes were tested at the pathway level. Using this strategy, this study identified two transmembrane transporter activity-related pathways that influenced serum urate levels and the pathogenesis of gout.

## Results

### Pathway analysis for serum urate in GWAS datasets

The pathway analysis of the GWAS data from European and US populations identified nine candidate causal genes (*PKD2*, *SLC2A9*, *SLC17A3*, *SLC22A11*, *SLC17A1*, *SLC17A4*, *SLC5A6*, *SLC17A2* and *SLC16A9*) and two candidate causal pathways (the ion transmembrane transporter activity pathway (GO: 0015075) and the secondary active transmembrane transporter activity pathway (GO: 0015291)) associated with serum urate concentration (*P*_FDR_ = 0.004 and 0.007) (Table 1). Among the nine candidate genes, *SLC17A2* and *SLC5A6* were two novel genes associated with serum urate concentration (Table 1 and Table S1). The nine candidate genes were distinctly clustered in the two candidate causal pathways, and the genes of the two pathways revealed some overlap. For example, *SLC2A9*, *SLC17A3*, *SLC22A11*, *SLC17A1*, *SLC17A4*, *SLC5A6* and *SLC17A2* were involved in both the two pathways, while *PKD2* and *SLC16A9* were only involved in one pathway each (Table 1). Additional information about the differences and the commonalities of the genes in the two candidate causal pathways are shown in Table S2.

**Table 1:** Candidate causal pathways of serum urate identified by GWAS data.

Pathway	Function	Candidate genes	*P*	*P*_FDR_
GO:0015075	ion transmembrane transporter activity	*PKD2*, *SLC2A9*, *SLC17A3*, *SLC22A11*, *SLC17A1*, *SLC17A4*, *SLC5A6*, *SLC17A2*	<0.001	0.004
GO:0015291	secondary active transmembrane transporter activity	*SLC2A9*, *SLC17A3*, *SLC22A11*, *SLC17A1*, *SLC17A4*, *SLC5A6*, *SLC16A9*, *SLC17A2*	<0.001	0.007

*P*_FDR_ means *P* value adjusted for multiple comparisons correction using FDR method.

### Candidate causal pathways associated with serum urate and gout

To verify the association between the two candidate pathways and the serum urate concentration in the Chinese population and to further analyze their effects on the pathogenesis of gout, approximately 5,000 Chinese individuals were enrolled in this study. Four candidate genes (*PKD2*, *SLC2A9*, *SLC17A3* and *SLC17A1*) were verified to be associated with serum urate with *P*_FDR_ values less than 0.05 (Table 2). However, *SLC17A2*, *SLC16A9*, *SLC22A11*, *SLC17A4* and *SLC5A6* were not associated with serum urate concentration in the Chinese population, suggesting that ethnicity might be a heterogeneity factor for the associations. Both the two candidate pathways were confirmed to affect the concentration of serum urate (GO: 0015075, *P*_FDR_ = 1.88E-05; GO: 0015291, *P*_FDR_ = 3.44E-04, respectively).

**Table 2:** Association between genetic variants in pathways and serum urate, hyperuricemia and gout.

Variants	Function	Gene	Effect allele	MAF		_Serum Urate_			_Gout vs. HUA_			_Gout vs. Control_		
						β	*P*	*P*_FDR_	OR	*P*	*P*_FDR_	OR	*P*	*P*_FDR_
**SNP**														
rs2728121	3utr	*PKD2*	C	0.45	Male	−11.09	2.67E-06	**2**.**40E-05**	0.81	7.53E-03	0.068	0.67	8.50E-09	**7**.**65E-08**
					Female	−12.52	0.011	0.101	0.70	0.250	1.000	0.55	0.047	0.421
					Total	−11.18	1.31E-07	**1**.**18E-06**	0.81	2.95E-03	**0**.**027**	0.66	7.51E-11	**6**.**76E-10**
rs13129697	intron	*SLC2A9*	T	0.47	Male	10.11	1.46E-05	**6**.**55E-05**	1.01	0.908	0.908	1.20	0.011	**0**.**019**
					Female	9.98	0.026	0.079	1.08	0.884	0.995	1.22	0.572	1.000
					Total	10.43	9.56E-06	**4**.**30E-05**	1.01	0.888	0.888	1.19	9.19E-03	**0**.**017**
rs2242206	missense	*SLC16A9*	G	0.40	Male	−0.64	0.921	0.921	1.12	0.130	0.235	1.10	0.201	0.258
					Female	8.82	0.069	0.155	1.22	0.563	1.000	1.54	0.150	0.675
					Total	2.03	0.407	0.524	1.09	0.242	0.364	1.12	0.068	0.101
rs1165165	missense	*SLC17A3*	T	0.14	Male	7.27	0.045	0.081	0.90	0.339	0.436	0.99	0.960	0.960
					Female	14.43	0.017	0.079	0.81	0.843	1.000	0.93	1.000	1.000
					Total	9.24	3.09E-03	**9**.**26E-03**	0.90	0.286	0.368	1.00	0.963	0.963
rs3759053	5upstream	*SLC22A11*	T	0.06	Male	5.64	0.171	0.256	1.04	0.768	0.864	1.23	0.131	0.197
					Female	10.21	0.340	0.437	0.77	1.000	1.000	0.96	1.000	1.000
					Total	7.11	0.101	0.151	0.94	0.733	0.825	1.14	0.350	0.394
rs1165196	missense	*SLC17A1*	G	0.19	Male	−8.75	3.88E-03	**0**.**012**	0.80	0.046	0.104	0.68	5.07E-05	**2**.**28E-04**
					Female	−5.78	0.585	0.585	1.09	0.856	1.000	1.19	0.589	1.000
					Total	−7.31	6.24E-03	**0**.**014**	0.79	0.018	**0**.**041**	0.71	1.48E-04	**6**.**66E-04**
rs11754288	missense	*SLC17A4*	A	0.17	Male	−5.92	0.044	0.100	0.79	0.031	0.094	0.69	2.38E-04	**7**.**15E-04**
					Female	−5.77	0.508	0.572	0.51	0.179	1.000	0.53	0.253	0.760
					Total	−5.72	0.046	0.083	0.77	9.85E-03	**0**.**030**	0.71	1.72E-04	**5**.**15E-04**
rs1395	missense	*SLC5A6*	G	0.13	Male	−1.68	0.693	0.780	0.88	0.276	0.414	0.89	0.318	0.357
					Female	7.83	0.145	0.260	0.57	0.317	0.951	0.75	0.672	1.000
					Total	1.72	0.697	0.784	0.79	0.038	0.068	0.87	0.160	0.206
rs2071299	missense	*SLC17A2*	G	0.32	Male	−1.41	0.549	0.706	0.80	8.76E-03	**0**.**039**	0.79	2.17E-03	**4**.**88E-03**
					Female	3.77	0.182	0.273	0.92	0.880	1.000	1.05	0.880	1.000
					Total	0.73	0.882	0.882	0.81	5.17E-03	**0**.**023**	0.83	7.60E-03	**0**.**017**
**Pathway**														
GO:0015075					Male		**1**.**92E-04**	**3**.**84E-04**		0.208	0.416		**6**.**12E-07**	**1**.**22E-06**
					Female		0.269	0.538		1.000	1.000		1.000	1.000
					Total		**9**.**42E-06**	**1**.**88E-05**		0.079	0.158		**5**.**41E-09**	**1**.**08E-08**
GO:0015291					Male		**5**.**24E-04**	**5**.**24E-04**		0.277	0.277		**1**.**83E-03**	**1**.**83E-03**
					Female		0.317	0.317		1.000	1.000		1.000	1.000
					Total		**3**.**44E-04**	**3**.**44E-04**		0.111	0.111		**2**.**66E-03**	**2**.**66E-03**

MAF, minor allele frequency in controls. HUA, hyperuricemia. β values for SNP in serum urate were calculated by linear regression adjusted age and gender. *P* values for SNP in serum urate were calculated by deviance analysis for linear regression adjusted age and gender. *P* values for SNP in hyperuricemia and gout were calculated by Fisher’s exact test. *P*_FDR_ value for SNPs and pathways were multiple corrected by FDR method. *P* values for pathway were calculated by Simes test.

In addition, five genes (*PKD2*, *SLC2A9*, *SLC17A1*, *SLC17A4* and *SLC17A2*) were determined to influence the risk of gout (all *P*_FDR_ < 0.05) (Table 2). To avoid the heterogeneity of gender, logistic regression adjusted for gender was performed and the result also showed that those five genes exhibited effects on the susceptibility of gout (all *P*_FDR_ < 0.05). *SLC17A2* was a novel gene that was found to be associated with the pathogenesis of gout (OR = 0.83, *P*_FDR_ = 0.017). *SLC17A4* was also identified as a candidate gene for gout risk (OR = 0.71, *P*_FDR_ = 5.15E-04), which was consistent with the result of our previous study^17^. In addition, two serum urate-associated pathways were found to affect the risk of gout (GO: 0015075, *P*_FDR_ = 1.08E-08; GO: 0015291, *P*_FDR_ = 2.66E-03, respectively), indicating a key role for elevated uric acid in the development of gout. To the best of our knowledge, this is the first time that these two candidate pathways have been associated with gout risk. To further understand the pathogenic mechanism linking serum urate and gout, we studied the genetic effects in transition process from hyperuricemia to gout. The result showed that *PKD2*, *SLC17A1*, *SLC17A4* and *SLC17A2* were associated with this pathogenesis (all *P*_FDR_ < 0.05) (Table 2), while the two candidate pathways had no effect on pathogenesis in the pathway analysis (GO: 0015075, *P*_FDR_ = 0.158; GO: 0015291, *P*_FDR_ = 0.111, respectively).

As expected, gender was a heterogeneity factor for serum urate and gout^18–20^ that affected the associations identified in the present study. Distinctly different results for those associations were observed between males and females (Table 2). For example, the two candidate pathways influenced the concentration of serum urate and gout risk in males (serum urate: GO: 0015075, *P*_FDR_ = 3.84E-04; GO: 0015291, *P*_FDR_ = 5.24E-04, gout: GO: 0015075, *P*_FDR_ = 1.22E-06; GO: 0015291, *P*_FDR_ = 1.83E-03, respectively), but not in females (serum urate: GO: 0015075, *P*_FDR_ = 0.538; GO: 0015291, *P*_FDR_ = 0.317, gout: GO: 0015075, *P*_FDR_ = 1.000; GO: 0015291, *P*_FDR_ = 1.000, respectively).

### Associations between candidate pathways and serum urate in the subgroups of BMI

Obesity is another heterogeneity factors that have been shown to influence serum urate concentration^21–24^; however, the effects of obesity on the associations between the candidate pathways and urate concentration were limited. Therefore, further analyses of subgroups of individuals divided by BMI was performed.

Based on the analysis of the BMI subgroups, four genes (*PKD2*, *SLC17A3*, *SLC17A1* and *SLC17A4*) were found to be associated with serum urate concentration in individuals with normal weight (18.5 ≦ BMI < 25) with a *P*_FDR_ less than 0.05 and two genes (*PKD2* and *SLC2A9*) were found to be associated with serum urate concentration in overweight subjects (BMI ≥ 25) (Table 3). In addition, the ion transmembrane transporter activity pathway (GO: 0015075) affected the concentrations of serum urate in both normal and overweight people (*P*_FDR_ = 0.018 and *P*_FDR_ = 2.15E-03, respectively), while the secondary active transmembrane transporter activity pathway (GO: 0015291) only affected the concentration of serum urate in overweight individuals with a *P*_FDR_ equal to 2.15E-03.

**Table 3:** Association between genetic variants in pathways and serum urate in subgroups of BMI.

Subgroup	Variants	Gene	Effect allele	Underweight			Normal			Overweight		
				β	*P*	*P*_FDR_	β	*P*	*P*_FDR_	β	*P*	*P*_FDR_
**BMI**	**SNP**											
	rs2728121	*PKD2*	C	−13.03	0.636	0.954	−10.98	1.29E-04	**1**.**16E-03**	−9.49	3.46E-03	**0**.**016**
	rs13129697	*SLC2A9*	T	21.05	0.235	0.424	8.98	0.034	0.061	12.82	2.98E-05	**2**.**68E-04**
	rs2242206	*SLC16A9*	G	−17.47	0.202	0.455	1.38	0.793	0.793	1.17	0.562	0.722
	rs1165165	*SLC17A3*	T	24.86	0.062	0.561	11.08	0.015	**0**.**044**	8.96	0.051	0.152
	rs3759053	*SLC22A11*	T	−9.15	0.666	0.856	11.46	0.139	0.209	4.08	0.307	0.553
	rs1165196	*SLC17A1*	G	−24.38	0.096	0.287	−11.65	3.20E-03	**0**.**014**	−3.05	0.449	0.673
	rs11754288	*SLC17A4*	A	−28.94	0.089	0.400	−9.99	0.016	**0**.**035**	−2.18	0.712	0.712
	rs1395	*SLC5A6*	G	1.26	0.990	0.990	−2.48	0.546	0.702	6.86	0.160	0.360
	rs2071299	*SLC17A2*	G	−1.79	0.865	0.973	−0.52	0.784	0.882	2.33	0.613	0.690
	**Pathway**											
	GO:0015075				0.990	0.990		**0**.**009**	**0**.**018**		**2**.**15E-03**	**2**.**15E-03**
	GO:0015291				0.898	1.000		0.115	0.115		**2**.**15E-03**	**2**.**15E-03**

Subgroup of BMI: 1, Underweight (BMI < 18.5); 2, Normal (18.5 ≦ BMI < 25); 3, Overweight (BMI ≧ 25). β values for SNP in serum urate were calculated by linear regression adjusted age and gender. *P* values for SNP in serum urate were calculated by deviance analysis for linear regression adjusted age and gender. *P* values for pathway were calculated by Simes test. *P*_FDR_ values for SNPs and pathways were multiple corrected by FDR method.

### The contribution of genetic effects on the pathogenesis of gout

**Figure 1:**
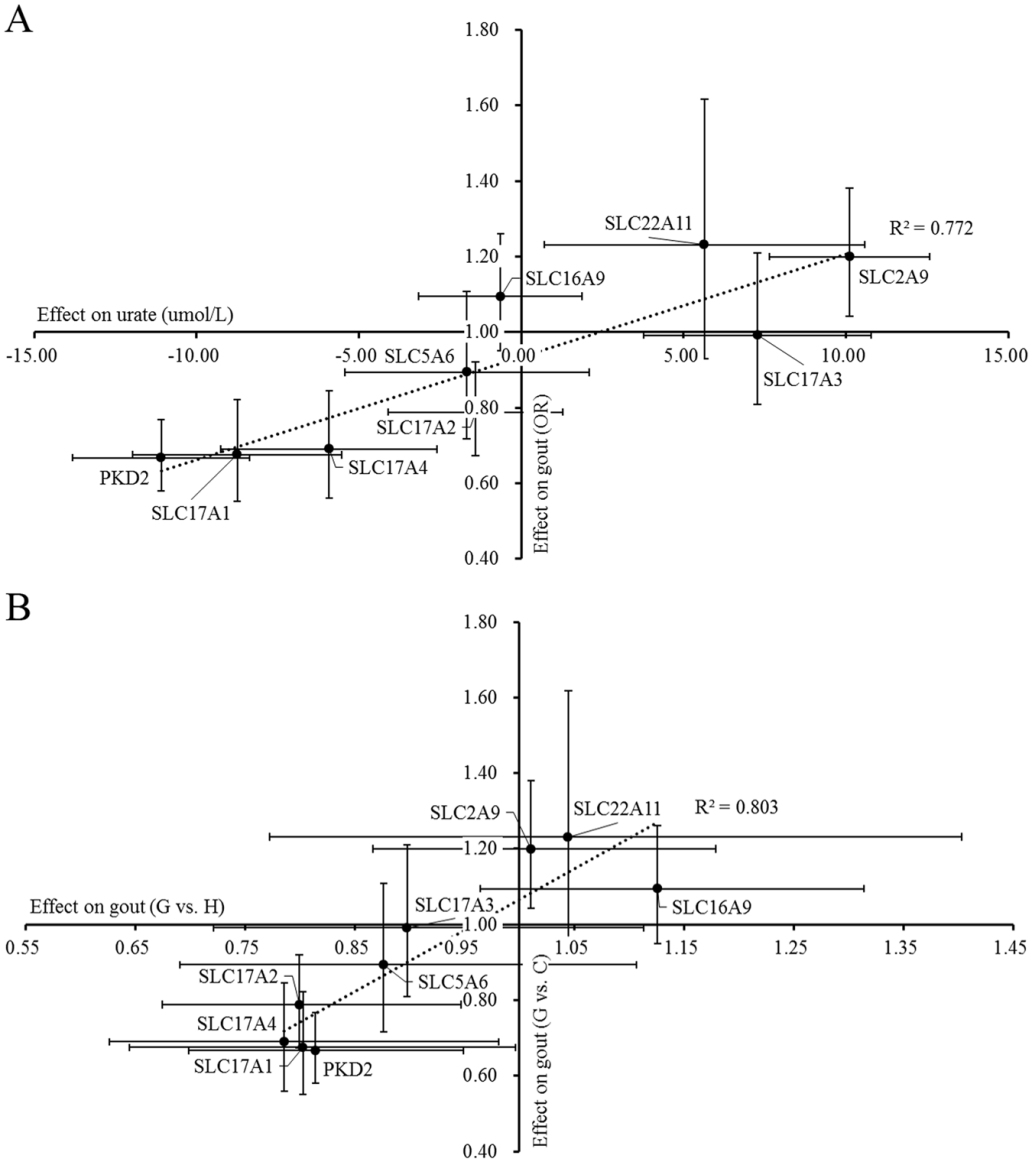
Positive correlation between the genetic effect on serum urate concentration and gout in males. (**A**) urate beta coefficients and gout odds ratios; (**B**) odds ratios for gout vs. hyperuricemia and gout odds ratios. Each confidence interval for the beta coefficient or odds ratio estimate was plotted as a bar of the point.

Because all candidate loci and pathways only influenced the concentrations of serum urate and the risk of gout in males (Table 2), further analysis for the candidate loci and pathways only preformed in males. Across the nine candidate loci for nine genes in the two pathways (rs2728121, rs13129697, rs2242206, rs1165165, rs3759053, rs1165196, rs11754288, rs1395 and rs2071299), for each additional effect allele in males, the genetic effect on serum urate was positively linearly correlated with the odds ratio for gout (R^2^ = 0.772) (Fig. 1A), which was consistent with the fact that high serum urate concentration is a key risk factor for the development of gout^17^. To measure the contribution of the development from hyperuricemia to gout, we analyzed the correlation between the genetic effects on the associations of gout and hyperuricemia/control. The result showed a high and linear correlation between those two combinations (R^2^ = 0.803), indicating the importance of this development in the pathogenesis of gout (Fig. 1B).

### Weight and unweighted genetic urate risk score of candidate pathways associated with hyperuricemia and gout

**Figure 2:**
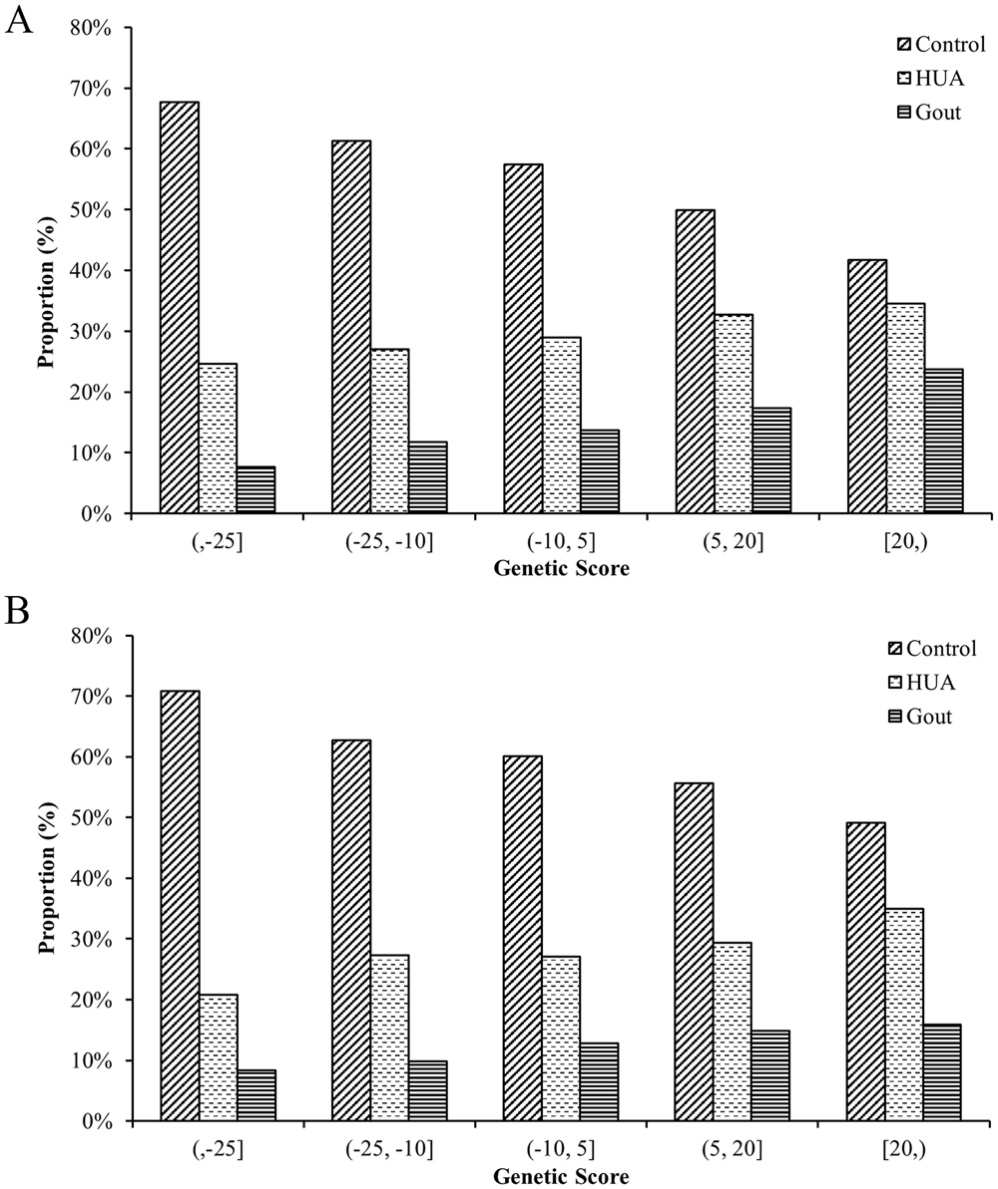
Relation between weighted genetic urate score and the proportions of hyperuricemia or gout patients in male subjects. (**A**) The ion transmembrane transporter activity pathway (GO: 0015075); (**B**) the secondary active transmembrane transporter activity pathway (GO: 0015291).

The weighted and unweighted genetic urate score (GRS) for candidate loci in the ion transmembrane transporter activity pathway (GO: 0015075) explained an average of 1.47% and 0.96% of the variance in serum urate concentration and was strongly associated with hyperuricemia and gout in males (weighted GRS: coefficients = 0.014, *P* = 2.53E-07; coefficients = 0.025, *P* = 2.14E-07, respectively; unweighted GRS: coefficients = 0.081, *P* = 4.27E-05; coefficients = 0.172, *P* = 1.49E-06, respectively). The weighted and unweighted GRS for the secondary active transmembrane transporter activity pathway (GO: 0015291) explained 0.81% and 0.45% of the variance in serum urate concentration and was found to affect hyperuricemia and gout in males (weighted GRS: coefficients = 0.013, *P* = 1.21E-04; coefficients = 0.016, *P* = 4.09E-03, respectively; unweighted GRS: coefficients = 0.059, *P* = 0.003; coefficients = 0.085, *P* = 0.013, respectively). Furthermore, the increased genetic urate score for the two candidate pathways resulted in elevated hyperuricemia and gout in males (Fig. 2). The above results suggested that the two candidate pathways influenced the pathogenesis of gout by modifying the level of serum urate.

### The different expression levels of candidate genes among the groups

**Figure 3:**
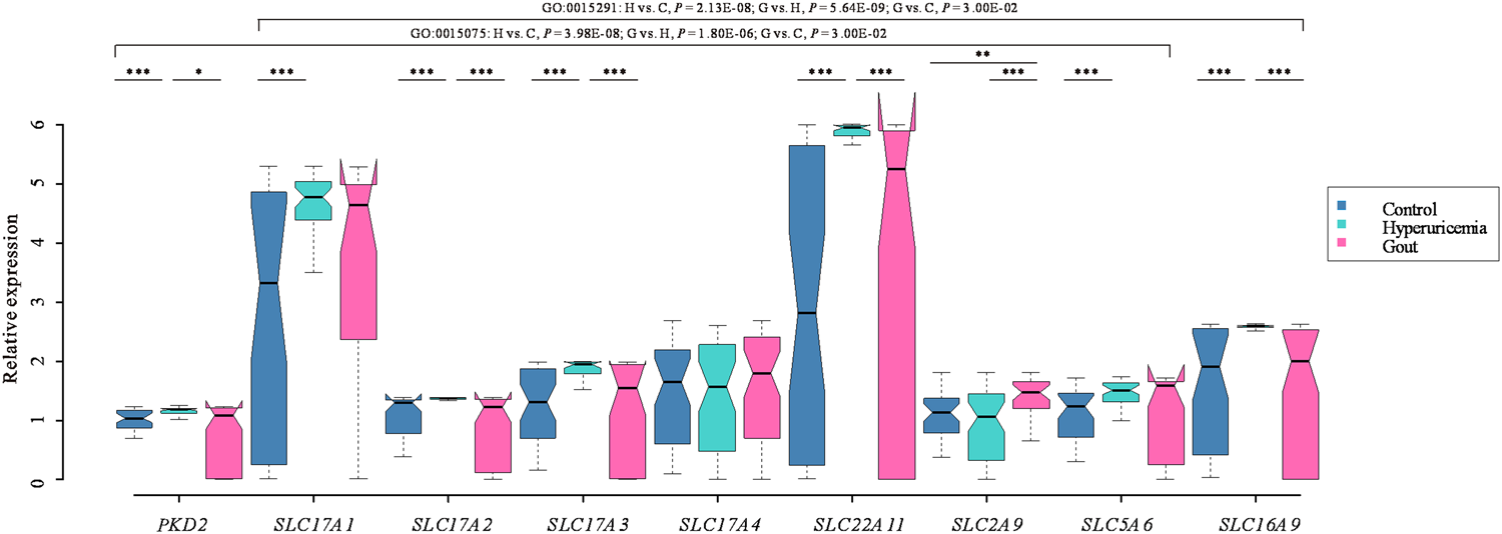
The differences in the expression of candidate genes at the pathway level among the groups. To analyze the transcription levels of candidate genes in pathway levels, we integrated the data of three genes (*SLC17A1*, *SLC17A4* and *SLC2A9*) mRNA expression that had been tested in our pervious study^17^ and the transcription levels of the other six candidate genes were analyzed in the present study. All those mRNA expression data of candidate genes come from the same samples. Because all candidate loci and pathways only influenced the concentrations of serum urate and the risk of gout in males, mRNA expressions of the candidate genes were only measured and analyzed in males. Quantitative polymerase chain reaction (qPCR) using SYBR Green was performed to test the relative mRNA levels of the candidate genes. The relative expression levels were analyzed using the Wilcoxon rank sum test. The data are illustrated as box plots. The upper edges and lower edges of the boxes represent the 75th percentiles and 25th percentiles, respectively. The lines inside the boxes represent the median of the data.

**Figure 4:**
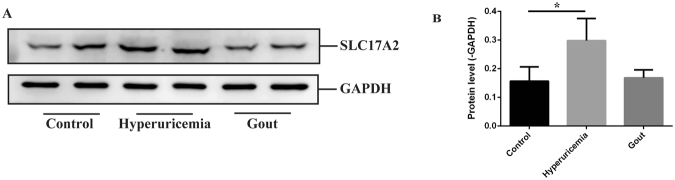
The protein levels of SLC17A2 were determined by western blot and analyzed by densitometry. (**A**) western blot analysis of SLC17A2 protein content in control, hyperuricemia, gout groups and (**B**) densitometry analysis of western blots for SLC17A2. GAPDH was used as an internal control. The full-length gels are presented in Supplementary Figures 1 and 2. Data are presented as mean ± SD of five samples and compared with Student’s t test; *P < 0.05, **P < 0.01.

To further validate the results described above, we analyzed the differences in transcription levels of the candidate genes in 70 male gout patients, 85 male hyperuricemia patients and 58 male healthy individuals (Fig. 3). Six candidate genes (*SLC17A1*, *SLC17A4* and *SLC2A9* were not under consideration in this section of result due to their mRNA expressions had been studied in our previous study^17^) in the two pathways, showed at least one significant difference in relative expression between the groups, suggesting that these loci may influence the risk of hyperuricemia and gout through alterations in their relative expression levels. In addition, as shown in Fig. 3, the mRNA expression levels were further analyzed at the pathway level and results showed significantly difference between the pairwise comparisons of gout patients, hyperuricemia patients and healthy controls. Regarding *SLC17A2*, its protein levels were measured among gout patients, hyperuricemia patients and healthy controls by performing Western blot (Figs 4, S1 and S2). The result showed the SLC17A2 protein level was higher in hyperuricemia patients than in others, which was consistent with our previous result in mRNA level (Fig. 3).

## Discussion

In the present study, we analyzed the genetic variants in candidate pathways affecting the concentration of serum urate and the risk of gout, and we identified two candidate pathways. All candidate loci and pathways involved in the present studies were analyzed in detail, including the analysis of associations in subgroups based on gender and body mass index. For each effect allele of the candidate genes, the genetic effect on uric acid was positively linearly correlated with the gout odds ratio. The expression of candidate genes was also analyzed at pathway level.

Nine candidate causal genes (*PKD2*, *SLC2A9*, *SLC17A3*, *SLC22A11*, *SLC17A1*, *SLC17A4*, *SLC5A6*, *SLC17A2* and *SLC16A9*) and two candidate causal pathways (the ion transmembrane transporter activity pathway (GO: 0015075) and the secondary active transmembrane transporter activity pathway (GO: 0015291)) were found to be associated with serum urate concentration in the pathway analysis of the GWAS dataset. Among these genes, *SLC17A2* and *SLC5A6* were two novel genes found to be associated with serum urate concentration in European and US individuals; however, this association was not replicated in the Chinese population, illustrating the heterogeneity effect of ethnicity on those associations^19,25^. However, the transcription levels of both *SLC17A2* and *SLC5A6* were significantly different between the hyperuricemia patients and healthy subjects, suggesting that these two genes potentially affect serum urate levels.

The protein coded by *SLC5A6*, sodium dependent multivitamin transporter (SMVT), is a transmembrane protein that mediates the cellular uptake of biotin, lipoic acid and pantothenic acid^26,27^. Biotin is an important cofactor for several carboxylase enzymes that are involved in the metabolism of carbohydrates, amino acids, and fatty acids^28^. Pantothenic acid is an essential component for the synthesis of coenzyme A, which is required for cellular metabolism^28^ and plays an important role in the tricarboxylic acid cycle (TCA cycle). Tricarboxylic acid cycle intermediates serve as substrates for purine synthesis, and purine metabolism can directly influence the concentration of serum urate. Taken together, *SLC5A6* might affect the concentrations of serum urate by mediating the cellular uptake of biotin, lipoic acid and pantothenic acid.

SLC17A2 (NPT3) belongs to a functional subgroup of the SLC17 family, type I phosphate transporters, which also includes SLC17A1, SLC17A3 and SLC17A4^29–33^. SLC17A1-4 are only found in mammals and originated from a common ancestor^34^, suggesting a similar urate transporter function of these four genes. A recent study in mouse showed that NPT3 transports urate was similar with other NPTs^35^. In addition, *SLC17A2* was expressed in the liver and the kidney^34,35^, which are important organs in the regulation of serum urate concentration. Urate is synthesized predominantly in the liver, and around two-thirds of urate excretion occurs via the kidneys^23^. In the present study, *SLC17A2* was found to be associated with gout risk either in the presence of hyperuricemia or not, and it variant played a protective role against gout. The common variant rs2071299 is an expression associated SNP and *SLC17A2* mRNA expression is modified by different genotypes of this variant. Based on the prediction of functional effects of this variant by F-SNP, rs2071299 can cause frameshift coding and splice site change^36^. In our study, the mRNA expression and protein levels of *SLC17A2* between healthy individuals and hyperuricemia patients were significantly different, also suggesting potential correlation between this variant and mRNA expression. Thus, *SLC17A2* might influence the development of gout by changing its expression.

Both the two candidate pathways identified in the present pathway analysis of GWAS data from the European and US population were found to affect the concentration of serum urate in the Chinese population. The function of the two candidate pathways involved transmembrane transporter activity, and several genes in these pathways had been identified to be urate transporters, such as *SLC2A9*, *SLC17A1* and *SLC17A4*^12,30,32^. However, another famous urate transporter, ABCG2, was not found in the two identified pathways, indicating that additional pathways that function in urate transport should be identified in future studies. Because elevated serum urate is a key risk factor for gout, we analyzed the associations between the two urate-associated pathways and gout, and we identified significant associations. In addition, the mRNA expression of candidate genes analyzed at pathway level also showed significant differences. To the best of our knowledge, this is the first time these two pathways have been associated with the pathogenesis of gout.

Heterogeneity factors, such as gender and BMI, can influence the associations of genetic variants and serum urate/gout, as reported in our previous study^17^. In the present study, the two candidate pathways played important roles in regulating the concentration of serum urate and in the development of gout in males, but not in females, which was consistent with the different effects of genetic variants between males and females^19^. Regarding BMI, the ion transmembrane transporter activity pathway affected the concentration of serum urate in both normal and overweight individuals, while the secondary active transmembrane transporter activity pathway only affected the concentration of serum urate in overweight subjects, suggesting that those associations were strongly influenced by BMI.

There are several limitations that should be considered in the present study. First, the number of female gout patients was limited. Second, the two candidate pathways identified above could only explain a portion of the variance of serum urate concentrations, and more urate associated-pathways should be identified in future studies. Finally, although gender and BMI were assessed in this study, some other heterogeneity factors associated with urate and gout were not considered. Therefore, further studies using pathway analysis should be performed.

Taken together, the present study identified the ion transmembrane transporter activity pathway (GO: 0015075) and the secondary active transmembrane transporter activity pathway (GO: 0015291) as associated with serum urate concentrations and the pathogenesis of gout. To the best of our knowledge, this is the first time these two pathways have been associated with gout. In addition, we identified a novel gout-associated gene, *SLC17A2*, in this study. These findings contributed to our understanding of the biological mechanisms regulating serum urate and gout on a pathway level and demonstrated the importance of analyzing GWAS data by pathway analysis.

## Materials and Methods

### Genome-wide association studies (GWASs) dataset

Serum urate-associated SNPs identified in two meta-analysis of genome-wide association studies (GWASs) for European and US population^8,14^ were analyzed in the present study. One study, comprised of 110,347 participants from 48 studies of serum urate concentrations, found that the median serum urate concentration was 5.2 mg/dl (the range was from 3.9 to 6.1 mg/dl)^8^. Another GWAS meta-analysis was performed in 5 population-based cohorts, which included 28,283 participants, for serum urate concentrations and found that the median (SD) of serum urate concentrations in the 5 cohorts were 354.8 µmol/l (96.3), 353.9 µmol/l (89.4), 327.4 µmol/l (85.7), 315.2 µmol/l (89.22), 321.2 µmol/l (80.9) and 324.3 µmol/l (117.2), respectively^14^. The descriptions of the GWASs included in this meta-analysis have been described in detail elsewhere^8,14^. The two studies described above were approved by the corresponding local ethics committees and written informed consent was obtained from the study participants.

### Pathway analysis for GWAS data

We used ICSNPathway^37^ to analyze GWAS datasets on serum urate concentrations. ICSNPathway offers a two-stage analysis for GWAS data. In the first stage, it pre-selects potential causal SNPs from the most significant SNPs in the GWAS dataset using linkage disequilibrium (LD) analysis and functional SNP annotation. In the second stage, it applies a pathway-based analysis (PBA) to identify the specific biological mechanisms for the pre-selected potential causal SNPs. Two GWAS datasets containing 1,279 and 2,201 SNPs associated with serum urate concentrations (*P* ≤ 4.00E-07 and *P* < 5.00E-08, respectively) were used as the inputs for the ICSNPathway analysis. The main parameters used in this study included: (a) threshold to extract the most significant SNPs: *P* value < 10^−6^; (b) HapMap population: European American (CEU) (HapMap 3); (c) LD cutoff: r^2^ > 0.8; (d) the maximum distance to search LD neighborhoods: 200 kb; (e) the method used for mapping a SNP to its corresponding gene (s): 20 kb upstream and downstream of the gene; (f) pathway set database: KEGG (http://www.genome.jp/kegg/pathway.html)^38^, BioCarta (http://www.biocarta.com/genes/index.asp), GO biological process (gene ontology, http://www.geneontology.org/)^39^ and GO molecular function (http://www.broadinstitute.org/gsea/msigdb/index.jsp)^40^; (g) number of genes in each pathway: minimum 5 and maximum 100; and (h) false discovery rate (FDR) cutoff for multiple testing correction for PBA: *P*_FDR_ < 0.05 was considered statistically significant.

### Participants

This study was approved by the Ethical Committees of the School of Life Sciences of Fudan University (approval number of 140) and was conducted in accordance with the guidelines and regulations of the Declaration of Helsinki. All participants provided written informed consent. A total of 582 gout patients who were clinically diagnosed according to the American College of Rheumatology diagnostic criteria^41^ were enrolled from Changhai Hospital, Taizhou People’s Hospital and Taixing People’s Hospital. An additional 4,332 healthy individuals without a history of gout were enrolled from the Taizhou Longitudinal Study^42^.

The 4,332 individuals were divided into subgroups of patients with hyperuricemia and healthy controls according to the threshold of serum urate deposition, i.e., approximately 417 µmol/L (equal to 7 mg/dl)^43^. Among the study population, 2,945 individuals with normal serum urate (≤417 µmol/L) were treated as healthy controls and 1,387 individuals with high serum urate measured on physical examination were considered hyperuricemia patients. In addition, the 4,332 individuals were also divided into subgroups according to body mass index (BMI) values following the categories of the World Health Organization^17,44^. Individuals were divided into three BMI subgroups (underweight: BMI < 18.5; normal weight: 18.50 ≤ BMI < 25; overweight: BMI ≥ 25) in this study. Overweight individuals contained those subjects considered obese with BMI greater than 30. The characteristics of the individuals in this study are shown in Table S3 and S4.

### RNA Isolation, cDNA Synthesis, and Real-time qPCR

To analyze the transcription levels of candidate genes in pathway level, we integrated the mRNA expression data of three genes (*SLC17A1*, *SLC17A4* and *SLC2A9*) that had been measured in our pervious study^17^ and the transcription levels of the other six candidate genes were analyzed in the present study. All those mRNA expression data of candidate genes come from the same samples. Randomly collected RNA from 70 male gout patients, 85 male hyperuricemia patients and 58 male healthy individuals was analyzed. RNA was extracted from blood cells using TRIzol reagent following the manufacturer’s instructions (Invitrogen, Carlsbad, CA, USA). Complementary DNA (cDNA) was synthesized by RNA reverse transcription using the High Capacity cDNA Reverse Transcription Kit (Applied Biosystems, Foster City, CA, USA) following the manufacturer’s protocol. Real-time quantitative polymerase chain reaction (qPCR) was performed using SYBR Premix Ex Taq (TaKaRa Biotech, Tokyo, Japan) with an ABI Prism 7900 Detector System (Applied Biosystems). The data obtained from the assays were analyzed by SDS 2.3 software (Applied Biosystems). The human housekeeping gene glyceraldehyde-3-phosphate dehydrogenase (*GAPDH*) was used as an internal control.

### Target SNPs selection

Based on pathway analysis in GWAS data, potential SNPs for candidate genes in pathway were selected. All potential SNPs were annotated by SNPnexus^45^ (http://www.snp-nexus.org/). And then the SNPs filtered by their SNP location and evaluated by the requirements of genotyping technology SNPscan. SNP that located at intergenic region and/or did not satisfy the requirements were replaced by other SNP in the same gene. Finally, after filtration, nine SNPs were chosen from the candidate genes.

### Weighted and unweighted genetic urate risk score analysis

To analyze the cumulative effects of the genetic variants in each pathway associated with serum urate, both weighted and unweighted genetic urate risk score (GRS) analysis were applied. In weighted GRS, for each locus, we multiplied the number of effect alleles (defined in Table 2) each male individual carried (0–2) by the beta coefficient from association analysis in males and then added the results to obtain a genetic urate risk score for one pathway^14^. The equation for the genetic urate risk score for the ion transmembrane transporter activity pathway (GO: 0015075) was as follows: rs2728121(N) x beta + rs1165165(N) x beta + rs1165196(N) x beta + rs11754288(N) x beta + rs13129697(N) x beta + rs1395(N) x beta + rs2071299(N) x beta + rs3759053(N) x beta; the equation for the genetic urate risk score for the secondary active transmembrane transporter activity pathway (GO: 0015291) was as follows: rs2242206(N) x beta + rs1165165(N) x beta + rs1165196(N) x beta + rs11754288(N) x beta + rs13129697(N) x beta + rs1395(N) x beta + rs2071299(N) x beta + rs3759053(N) x beta, where N of each locus denotes the number of copies of that allele carried by each male individual and the beta value represents the effect size per allele in serum urate concentration in males. The unweighted GRS was calculated by summing the number of effect alleles across the variants in pathways with identical risk. The associations between weight/unweighted GRS and hyperuricemia/gout in males were tested by logistic regression with adjustment for age. A linear regression was used to analyze the relationship between weight/unweighted GRS and the serum urate concentration in males.

### Immunoblotting analysis

In the study, five individuals in each of three groups (gout patient, hyperuricemia patient and healthy control) were enrolled from Changhai Hospital in Shanghai and their blood samples were collected to perform Western blotting analysis. All participants signed the written informed consent form. PBMCs were extracted by density-gradient centrifugation (Ficoll-PaqueTM PLUS; GE Healthcare Bio, Chicago, IL, USA). And then the cell lysates from the PBMCs were applied for Western blotting analysis in this study. Equal amounts of protein from samples were subjected to 12% SDS PAGE after which protein was transferred to PVDF membranes (Millipore, Billerica, MA, USA). Membranes were blocked in 5% milk in TBST at room temperature for 2 h after which they were incubated with one of the following primary antibodies: rabbit polyclonal to SLC17A2 (1:1000) (Abcam, Hong Kong, Ltd.), and internal control GAPDH (1:3000–5000) (Vazyme, China) at 4 °C overnight. Membranes were washed three times with TBST for a total of 30 minutes and then incubated with the horse-radish peroxidase-conjugated secondary antibody of goat anti-rabbit, or anti-mouse lgG for 2 h at room temperature. The protein bands were visualized with ECL solution.

### Statistical analyses

The genotypes at each locus were evaluated for deviation from Hardy-Weinberg equilibrium. Associations with serum urate were tested by linear regression and deviance analysis for linear regression, with adjustments for gender and age. Fisher’s exact test in the additive model and logistic regression adjusted for gender were used to test the association between each locus and gout. Gender subgroups were used in the serum urate and gout analyses. Furthermore, subgroups of body mass index (BMI) was also used in the serum urate analysis. A nominal *P* value was calculated and was corrected for multiple testing using the FDR method (*P*_FDR_). *P*_FDR_ values less than 0.05 were considered statistically significant.

The associations between the pathways and the two phenotypes (serum urate and gout) were tested using the Simes test^46^. The Simes test improves upon the Bonferroni method by rejecting H0 for any *m* = 1, 2, …, *N*-1, *N*, *P* (*m*) ≤ *mα/N*, where *P* (1) ≤ … ≤ *P* (*N*), showing the ordered *P* values, with *P* (*n*) being the *n*th smallest *P* value. In our study, the *P* values were replaced with *P*_FDR_ values of the loci to control the false positive rate. In addition, the *P* value of the pathway was also multiple corrected using the FDR method (*P*_FDR_).

The mRNA expression data were visualized using box plots, and all outliers were removed. The differences between the different groups in the transcription levels of the candidate genes in the identified pathways were analyzed using the Wilcoxon rank sum test. The differences in gene expression at the pathway level were tested using the Simes test. A *P* value less than 0.05 was considered statistically significant.

All statistical analyses were performed using R (Version 3.0.2: www.r-project.org/).

## Electronic supplementary material

Supplementary files
