# Supplementary material for: Genetic variants in two pathways influence serum urate levels and gout risk: a systematic pathway analysis
Source: Sci Rep. 2018 Mar 1;8:3848. doi: 10.1038/s41598-018-21858-0 (PMC5832812; doi:10.1038/s41598-018-21858-0)
Supplement: Supplementary file 1 — Supplementary files [file 41598_2018_21858_MOESM1_ESM.pdf]

Genetic variants in two pathways influence serum urate levels and gout risk: a systematic pathway analysis

Zheng Dong[1#], Jingru Zhou[1#], Xia Xu[2#], Shuai Jiang[1#], Yuan Li[1], Dongbao Zhao[2], Chengde Yang[3], Yanyun Ma[1], Yi Wang[1], Hongjun He[4], Hengdong Ji[5], Juan Zhang[6], Ziyu Yuan[6], Yajun Yang[1,6], Xiaofeng Wang[1,6], Yafei Pang[2], Li Jin[1,6], Hejian Zou[7,8], Jiucun Wang[1, 6,8]

[1] State Key Laboratory of Genetic Engineering, Collaborative Innovation Center for Genetics and Development, School of Life Sciences, Fudan University Jiangwan Campus, Shanghai, China.

[2] Division of Rheumatology and Immunology, Changhai Hospital, Shanghai, China.

[3] Division of Rheumatology, Ruijin Hospital, Shanghai Jiaotong University School of Medicine, Shanghai, China.

[4] Division of Rheumatology, Taixing People's Hospital, Jiangsu Province, China.

[5] Division of Rheumatology, Taizhou People's Hospital, Jiangsu Province, China.

[6] Fudan-Taizhou Institute of Health Sciences, Taizhou, Jiangsu Province, China.

[7] Division of Rheumatology, Huashan Hospital, Fudan University, Shanghai, China.

[8] Institute of Rheumatology, Immunology and Allergy, Fudan University, Shanghai, China.

# These authors contributed equally to this work.

Address corresponding to Jiucun Wang, Ph.D, School of Life Sciences, Fudan University, 2005 Songhu Road, Shanghai 200438, People's Republic of China. (e-mail: jcwang@fudan.edu.cn).

Table S1. Candidate causal SNPs for serum urate in pathway analysis for GWAS data

SNP	Functional class	Gene	$-log_{10}(P)^a$	In LD with	$-log_{10}(P)^b$
rs2728121	regulatory_region	*PKD2*	33.066	rs2728121	33.066
rs942377	regulatory_region	*SLC17A1*	13.585	rs942377	13.585
rs3799346	regulatory_region	*SLC17A1*	13.569	rs3799346	13.569
rs3799344	regulatory_region	*SLC17A1*	19.854	rs3799344	19.854
rs12209856	regulatory_region	*SLC17A1*	12.62	rs12209856	12.62
rs9393670	regulatory_region	*SLC17A1*	12.569	rs9393670	12.569
rs9393671	regulatory_region	*SLC17A1*	12.569	rs9393671	12.569
rs2096386	regulatory_region	*SLC17A1*	9.886	rs2096386	9.886
rs1165215	regulatory_region	*SLC17A1*	24.229	rs1165215	24.229
rs1165196	non_synonymous_coding (deleterious)	*SLC17A1*	24.301	rs1165196	24.301
rs1165156	regulatory_region	*SLC17A1*	12.721	rs1165156	12.721
rs1165157	regulatory_region	*SLC17A1*	12.77	rs1165157	12.77
rs1165155	regulatory_region	*SLC17A1*	12.538	rs1165155	12.538
rs3923	regulatory_region	*SLC17A1*	10.509	rs3923	10.509
rs2762353	regulatory_region	*SLC17A1*	23.208	rs2762353	23.208
rs10214468	regulatory_region	*SLC17A1*	7.409	rs10214468	7.409
rs13197601	regulatory_region	*SLC17A1*	21.658	rs13197601	21.658
rs12662869	regulatory_region	*SLC17A1*	13.745	rs12662869	13.745
rs3757131	regulatory_region	*SLC17A1*	21.638	rs3757131	21.638
rs13213957	regulatory_region	*SLC17A1*	8.658	rs13213957	8.658
rs13200921	regulatory_region	*SLC17A1*	9.553	rs13200921	9.553
**rs2071299**	non_synonymous_coding	***SLC17A2***	-	rs1541987 ($r^2 = 0.92$)	19.42
rs1165165	non_synonymous_coding	*SLC17A3*	12.377	rs1165165	12.377
rs11754288	non_synonymous_coding	*SLC17A4*	48.027	rs11754288	48.027
rs13137343	regulatory_region	*SLC2A9*	86.959	rs13137343	86.959
rs16890979	non_synonymous_coding	*SLC2A9*	240.495	rs16890979	240.495
rs2280205	non_synonymous_coding	*SLC2A9*	21.569	rs2280205	21.569
**rs1395**	non_synonymous_coding (deleterious)	***SLC5A6***	7.538	rs1395	7.538
rs2242206	non_synonymous_coding	*SLC16A9*	13.824	rs2242206	13.824

a -log10(P) for candidate causal SNP in original GWAS. '-' denotes that this SNP is not represented in the original GWAS.

b -log10(P) for the SNP (which candidate causal SNP is in LD with) in original GWAS.

Rs2078267 in *SLC22A11* was mapped in pathways, but it local at intron region and was not considered causal SNP.

SLC17A2 and *SLC5A6* were shown in bold.

Table S2. Non-identified genes in the two candidate causal pathways through pathway analysis for GWAS data.

Genes in GO:0015075 pathway but not mapped with variant				Genes in GO:0015291 but not mapped with variant		Common genes in two candidate pathways
A0AVG2	NHEDC2	Q4G0N8	RYR2	A0AV02	SLC9A4	A2A2U1
A0PGH1	NMUR2	Q4G0S9	RYR3	A2A2U1	SLC9A5	A6NFI1
A0PJH2	NNT	Q4G0X4	S1PR3	A6NF70	SLC9A6	A6NGW4
A1DV59	NOLA1	Q4G129	SCL8A3	A6NFI1	SLC9A7	A6NKM8
A1X4P9	NOX1	Q4JCQ8	SCN10A	A6NGW4	SLC9A8	A8MPP9
A1X4Q0	NOX5	Q4KKU8	SCN11A	A6NIM6	SLC9A9	A8MSJ2
A2A2I8	NP_001003701	Q4QQG6	SCN1A	A6NKM8	SLCO1B1	A8MT54
A2A2U1	NP_001034663	Q4U2R8	SCN1B	A8MPP9	SLCO2B1	A8MUM6
A2A3F0	NP_001083060	Q4VAP7	SCN2A	A8MSJ2	TMCO3	A8MWM7
A2A3F1	NP_001106271	Q4VAQ5	SCN2B	A8MT54	XP_001720043	AE4
A2A3F3	NP_001119579	Q4VKI1	SCN3A	A8MUM6	XP_001721108	AKT1
A2A3F4	NP_055809	Q4VKI2	SCN3B	A8MWM7	XP_001722727	ANKH
A2A3F7	NP_060306	Q4VKI4	SCN4A	AE4	hCG_2015407	ASNA1
A2A3G9	NP_776297	Q4VNC0	SCN4B	AKT1	ncx1	B0S7E9
A2A3S1	NP_944493	Q4VNC1	SCN5A	ANKH		B1AL64
A2AAT0	NP_996827	Q4VXP0	SCN7A	ASNA1		B1AVM8
A2AVK2	NUDT9	Q4VXP2	SCN8A	B0S7E9		B3KS22
A2Q0V1	NUP153	Q4VXP3	SCN9A	B1AL64		B3KSM4
A3EY19	Nav1.5	Q4W595	SCNN1A	B1AVM8		B3KTU1
A3KN86	Nbla00402	Q4W5E3	SCNN1B	B3KS22		B3KTU2
A4D1R5	O00168	Q4ZG44	SCNN1D	B3KSM4		B3KVU0
A4FTX5	O00180	Q4ZJI4	SCNN1G	B3KTU1		DKFZp566I1346

A4IF51	O00244	Q504W2	SFXN1	B3KTU2	DKFZp686P10213
A5PKY2	O00299	Q504Y0	SFXN2	B3KVU0	DKFZp761D171
A5X5Y0	O00305	Q52LC2	SFXN3	CDH17	Em:AP000350.2
A6NC82	O00305-1	Q52PK6	SFXN4	DKFZp566I1346	ENSP00000216568
A6NCA9	O00337	Q52PU4	SFXN5	DKFZp686P10213	ENSP00000322354
A6NCB1	O00341	Q53ES0	SHKBP1	DKFZp761D171	ENSP00000350560
A6NCZ6	O00476	Q53FY0	SHROOM2	ENSP00000216568	ENSP00000351597
A6ND05	O00555	Q53HH2	SK3	ENSP00000322354	EPB3
A6ND09	O00555-2	Q53YR0	SLC10A1	ENSP00000350560	hCG_2015407
A6ND60	O00555-7	Q548A7	SLC10A2	ENSP00000351597	HIT000217122
A6NDN7	O00591	Q567R0	SLC10A3	EPB3	HIT000262994
A6NDV9	O00624	Q58I18	SLC10A4	Em:AP000350.2	LRCH1
A6NEF1	O14618	Q59F38	SLC10A5	HIT000217122	MORC3
A6NFI1	O14649	Q59F54	SLC10A6	HIT000262994	NAGLT1
A6NGW4	O14764	Q59G58	SLC10A7	LRCH1	NBC
A6NH10	O14791	Q59GA6	SLC11A2	MORC3	ncx1
A6NH75	O14863	Q59GJ7	SLC12A1	NAGLT1	NHEDC1
A6NKD4	O14949	Q59GK1	SLC12A2	NBC	NHEDC2
A6NKM8	O14957	Q59H29	SLC12A3	NHEDC1	NP_001106271
A6NLE7	O14983	Q59H69	SLC12A4	NHEDC2	NP_001119579
A6NLP4	O14983-2	Q5BLP4	SLC12A5	NP_001106271	NP_055809
A6NM02	O15244	Q5DP47	SLC12A6	NP_001119579	O00337
A6NM44	O15245	Q5F0I5	SLC12A7	NP_055809	O00341
A6NMB8	O15245-2	Q5G1L5	SLC13A2	O00337	O00476
A6NMJ0	O15245-4	Q5H9T8	SLC13A3	O00341	O00624

A6NN85	O15247	Q5HYC3	SLC13A4	O00476	O43511
A6NNF7	O15342	Q5I6N7	SLC15A1	O00624	O43681
A6NNJ6	O15399	Q5JPP8	SLC15A2	O15374	O43826
A6PVM7	O15431	Q5JQ20	SLC17A5	O15375	O43868
A6PVT7	O15432	Q5JQ21	SLC17A7	O15403	O60721
A6PVT8	O15438	Q5JRE4	SLC1A1	O15427	O76082
A6QRJ1	O15439	Q5JSD1	SLC1A2	O43511	O94956
A7BJ74	O15440	Q5JSD2	SLC1A3	O43681	O95436
A7E2D8	O15547	Q5JSM7	SLC1A4	O43826	O95528
A8IKD7	O15554	Q5JUK3	SLC1A5	O43868	OTTHUMP00000004471
A8K1F5	O43424	Q5JXL4	SLC1A6	O60669	OTTHUMP00000012508
A8K1Y4	O43448	Q5JXX5	SLC1A7	O60721	OTTHUMP00000012509
A8K519	O43497	Q5JXY1	SLC20A1	O60779	OTTHUMP00000025817
A8K7I4	O43511	Q5K3P7	SLC20A2	O76082	OTTHUMP00000025947
A8KAB9	O43520	Q5M8T2	SLC22A1	O94956	OTTHUMP00000025948
A8MPP9	O43525	Q5MJQ3	SLC22A13	O95436	OTTHUMP00000028645
A8MPS3	O43526	Q5PT55	SLC22A14	O95528	OTTHUMP00000030098
A8MPV7	O43636	Q5QNZ2	SLC22A2	O95907	OTTHUMP00000165211
A8MPY1	O43681	Q5SNW4	SLC22A3	OTTHUMP00000004471	OTTHUMP00000166146
A8MQ22	O43826	Q5SQ17	SLC22A4	OTTHUMP00000012508	OTTHUMP00000166149
A8MQ46	O43861	Q5SVJ5	SLC22A5	OTTHUMP00000012509	OTTHUMP00000167510
A8MQ47	O43868	Q5SVJ7	SLC22A6	OTTHUMP00000025817	OTTHUMP00000167512
A8MSJ2	O60312	Q5SVJ8	SLC22A7	OTTHUMP00000025947	OTTHUMP00000170920
A8MT31	O60359	Q5SVJ9	SLC22A8	OTTHUMP00000025948	OTTHUMP00000172223
A8MT54	O60391	Q5SVK0	SLC23A2	OTTHUMP00000028645	OTTHUMP00000172224

A8MT92	O60423	Q5SVK2	SLC24A1	OTTHUMP00000030098	OTTHUMP00000177496
A8MTZ5	O60721	Q5SVK5	SLC24A3	OTTHUMP00000115462	OTTHUMP00000181864
A8MUE4	O60741	Q5SXD5	SLC25A11	OTTHUMP00000115463	OTTHUMP00000198950
A8MUH2	O60840	Q5SXE0	SLC25A3	OTTHUMP00000165211	OTTHUMP00000198951
A8MUK9	O60928	Q5T047	SLC25A37	OTTHUMP00000166146	OTTHUMP00000199574
A8MUM6	O60939	Q5T1M3	SLC26A1	OTTHUMP00000166149	OTTHUMP00000200880
A8MUN4	O75110	Q5T4K2	SLC26A11	OTTHUMP00000167510	OTTHUMP00000200882
A8MVK1	O75185	Q5T4K3	SLC26A2	OTTHUMP00000167512	P02730
A8MVK5	O75311	Q5T4K4	SLC26A3	OTTHUMP00000170920	P04920
A8MWM7	O75348	Q5T556	SLC26A4	OTTHUMP00000172223	P08195
A8MWU7	O75751	Q5T5E6	SLC26A5	OTTHUMP00000172224	P09131
A8MX51	O75762	Q5T5E7	SLC26A6	OTTHUMP00000177496	P11166
A8MXF8	O75947	Q5T5Q4	SLC26A7	OTTHUMP00000181864	P11168
A8MYH4	O75964	Q5T5Q6	SLC26A8	OTTHUMP00000198950	P11169
A8MYL6	O76082	Q5T5Q7	SLC26A9	OTTHUMP00000198951	P13866
A8MYL9	O76090	Q5T7Q5	SLC28A1	OTTHUMP00000199574	P14672
A8MYU2	O94759	Q5TAB8	SLC28A2	OTTHUMP00000200880	P19634
A9Z1Z8	O94823	Q5TAH2	SLC28A3	OTTHUMP00000200882	P22732
AAD14	O94956	Q5TBB2	SLC2A1	P02730	P30531
ABCA1	O95069	Q5TBB3	SLC2A10	P04920	P31639
ABCB11	O95180	Q5TC02	SLC2A11	P08195	P31749
ABCC2	O95226	Q5TC05	SLC2A12	P09131	P32418
ABCC3	O95259	Q5TF39	SLC2A14	P11166	P40879
ABCC4	O95264	Q5TG78	SLC2A2	P11168	P43003
ABCC5	O95264-2	Q5TG79	SLC2A3	P11169	P43004

ABCC8	O95279	Q5TG80	SLC2A4	P12236	P43005
ACCN1	O95342	Q5TG81	SLC2A5	P13866	P43007
ACCN2	O95409	Q5TG84	SLC2A6	P14672	P46059
ACCN3	O95436	Q5U089	SLC2A7	P16260	P48065
ACCN4	O95477	Q5U5S6	SLC2A8	P19634	P48066
ACCN5	O95528	Q5UM68	SLC30A1	P22732	P48067
ADAMTS8	O95670	Q5V9X8	SLC30A10	P23975	P48664
AE4	O95833	Q5V9X9	SLC30A2	P30531	P48751
AKT1	O96008	Q5VSF4	SLC30A3	P31639	P48764
ANKH	OCA2	Q5VSF5	SLC30A4	P31641	P50443
APOL1	OCTN2VT	Q5VSF9	SLC30A5	P31645	P53794
ASNA1	ORAI1	Q5VTR3	SLC30A6	P31749	P55011
ATOX1	OTTHUMP00000004471	Q5VTU8	SLC30A7	P32418	P55017
ATP10A	OTTHUMP00000012508	Q5VVV3	SLC30A8	P36021	P57057
ATP10B	OTTHUMP00000012509	Q5VVV4	SLC30A9	P40879	P57103
ATP10D	OTTHUMP00000014143	Q5VVV5	SLC31A1	P41440	P58743
ATP11A	OTTHUMP00000016087	Q5VVV6	SLC31A2	P43003	P78381
ATP11B	OTTHUMP00000019906	Q5VVV7	SLC32A1	P43004	P78382
ATP11C	OTTHUMP00000021850	Q5VVV9	SLC34A1	P43005	P78383
ATP12A	OTTHUMP00000021852	Q5VVW0	SLC34A2	P43007	P78488
ATP13A1	OTTHUMP00000021853	Q5VVW5	SLC34A3	P46059	PEA15
ATP13A2	OTTHUMP00000022895	Q5VWQ3	SLC35A1	P48029	PRES
ATP13A3	OTTHUMP00000022951	Q5VWQ4	SLC35A2	P48065	Q00325
ATP13A4	OTTHUMP00000024162	Q5VX33	SLC35A3	P48066	Q02978
ATP13A5	OTTHUMP00000024163	Q5W1L7	SLC35A4	P48067	Q05BQ3

ATP1A1	OTTHUMP00000025807	Q5W1L8	SLC35A5	P48664	Q06495
ATP1A2	OTTHUMP00000025817	Q5Y190	SLC35B1	P48751	Q08357
ATP1A3	OTTHUMP00000025893	Q659C3	SLC35B4	P48764	Q0GE19
ATP1A4	OTTHUMP00000025899	Q659E4	SLC35C1	P50443	Q12835
ATP1B1	OTTHUMP00000025947	Q68CS5	SLC35D1	P53794	Q12908
ATP1B2	OTTHUMP00000025948	Q68DU8	SLC35D2	P53985	Q13032
ATP1B3	OTTHUMP00000028645	Q693B1	SLC35D3	P55011	Q13183
ATP1B4	OTTHUMP00000028797	Q695T7	SLC36A1	P55017	Q13621
ATP2A1	OTTHUMP00000030098	Q6A0F2	SLC36A2	P57057	Q13717
ATP2A2	OTTHUMP00000030507	Q6A0F3	SLC37A1	P57103	Q14916
ATP2A3	OTTHUMP00000034091	Q6AI14	SLC37A2	P58743	Q14940
ATP2B2	OTTHUMP00000041752	Q6EWN2	SLC37A3	P78381	Q14973
ATP2B3	OTTHUMP00000042062	Q6ICJ5	SLC37A4	P78382	Q15121
ATP2B4	OTTHUMP00000045936	Q6IPA9	SLC38A1	P78383	Q15758
ATP2C1	OTTHUMP00000045937	Q6IPQ3	SLC39A1	P78488	Q16348
ATP2C2	OTTHUMP00000066278	Q6JHX1	SLC39A10	PEA15	Q17R43
ATP4A	OTTHUMP00000072922	Q6JXY2	SLC39A11	PRES	Q17RM9
ATP4B	OTTHUMP00000072924	Q6L5N3	SLC39A12	Q00325	Q29RF8
ATP5A1	OTTHUMP00000072928	Q6LAJ8	SLC39A13	Q01959	Q2M1U9
ATP5B	OTTHUMP00000072971	Q6LAJ9	SLC39A14	Q02978	Q2TB63
ATP5C1	OTTHUMP00000073056	Q6MZM0	SLC39A2	Q05BQ3	Q2VI00
ATP5D	OTTHUMP00000073083	Q6MZW7	SLC39A3	Q06495	Q2Y0W8
ATP5E	OTTHUMP00000073084	Q6NT69	SLC39A4	Q08357	Q3KNW5
ATP5EP2	OTTHUMP00000073085	Q6NVJ2	SLC39A5	Q0GE19	Q495M3
ATP5F1	OTTHUMP00000076720	Q6NXT4	SLC39A6	Q12835	Q495M3-2

ATP5G1	OTTHUMP00000077683	Q6NXT4-2	SLC39A7	Q12864	Q495M3-3
ATP5G2	OTTHUMP00000080339	Q6P148	SLC39A8	Q12908	Q496J0
ATP5G3	OTTHUMP00000080853	Q6P1Z3	SLC39A9	Q13032	Q496J2
ATP5H	OTTHUMP00000096570	Q6P4A7	SLC3A2	Q13183	Q4G0N8
ATP5I	OTTHUMP00000096571	Q6P4C1	SLC40A1	Q13621	Q4JCQ8
ATP5J	OTTHUMP00000096573	Q6P5W5	SLC41A1	Q13717	Q4QQG6
ATP5J2	OTTHUMP00000096574	Q6PCC3	SLC41A2	Q14916	Q4U2R8
ATP5L	OTTHUMP00000115741	Q6PEL1	SLC41A3	Q14940	Q4ZJI4
ATP5L2	OTTHUMP00000115746	Q6PI47	SLC45A1	Q14973	Q504W2
ATP5O	OTTHUMP00000115747	Q6PIL6	SLC4A1	Q15121	Q548A7
ATP5S	OTTHUMP00000123432	Q6PIU1	SLC4A10	Q15758	Q59F54
ATP6	OTTHUMP00000147529	Q6PJI0	SLC4A11	Q16348	Q59GA6
ATP6AP1	OTTHUMP00000163795	Q6PML9	SLC4A2	Q17R43	Q59GJ7
ATP6G	OTTHUMP00000164642	Q6PN88	SLC4A3	Q17RM9	Q59GK1
ATP6V0A1	OTTHUMP00000164900	Q6PXP3	SLC4A4	Q1EHB4	Q5JPP8
ATP6V0A2	OTTHUMP00000165211	Q6TN97	SLC4A5	Q29RF8	Q5K3P7
ATP6V0A4	OTTHUMP00000165948	Q6U841	SLC4A7	Q2M1U9	Q5M8T2
ATP6V0B	OTTHUMP00000166146	Q6UVK1	SLC4A8	Q2TB63	Q5PT55
ATP6V0C	OTTHUMP00000166149	Q6UVM3	SLC5A1	Q2VI00	Q5SXD5
ATP6V0D1	OTTHUMP00000166187	Q6UWJ1	SLC5A11	Q2Y0W8	Q5SXE0
ATP6V0D2	OTTHUMP00000166188	Q6UXQ9	SLC5A2	Q3KNW5	Q5TAB8
ATP6V0E1	OTTHUMP00000166198	Q6X6Z3	SLC5A3	Q495M3	Q5TAH2
ATP6V0E2	OTTHUMP00000166367	Q6X6Z5	SLC5A4	Q495M3-2	Q5TBB2
ATP6V1A	OTTHUMP00000166981	Q6XR72	SLC5A5	Q495M3-3	Q5TBB3
ATP6V1B1	OTTHUMP00000167287	Q6YIQ9	SLC5A7	Q496J0	Q5TF39

ATP6V1B2	OTTHUMP00000167288	Q6ZMH5	SLC6A1	Q496J2	Q5U5S6
ATP6V1C1	OTTHUMP00000167289	Q6ZMM8	SLC6A11	Q4G0N8	Q5VVV3
ATP6V1C2	OTTHUMP00000167442	Q6ZMN3	SLC6A12	Q4JCQ8	Q5VVV4
ATP6V1D	OTTHUMP00000167444	Q6ZNA7	SLC6A13	Q4QQG6	Q5VVV5
ATP6V1E1	OTTHUMP00000167510	Q6ZNE4	SLC6A14	Q4U2R8	Q5VVV6
ATP6V1E2	OTTHUMP00000167512	Q6ZP48	SLC6A15	Q4ZJI4	Q5VVV7
ATP6V1F	OTTHUMP00000167722	Q6ZUB2	SLC6A16	Q504W2	Q5VVV9
ATP6V1G2	OTTHUMP00000167725	Q6ZUX8	SLC6A17	Q53S99	Q5VVW0
ATP6V1G3	OTTHUMP00000168048	Q6ZW95	SLC6A18	Q548A7	Q5VVW5
ATP6V1H	OTTHUMP00000168050	Q6ZWB6	SLC6A19	Q59EV7	Q5W1L7
ATP7A	OTTHUMP00000168051	Q708S9	SLC6A2	Q59F54	Q5W1L8
ATP7B	OTTHUMP00000168053	Q70T25	SLC6A20	Q59FC0	Q5Y190
ATP8	OTTHUMP00000168054	Q70T26	SLC6A5	Q59GA6	Q659E4
ATP8A1	OTTHUMP00000168679	Q70Z44	SLC6A7	Q59GJ7	Q695T7
ATP8A2	OTTHUMP00000169032	Q719H9	SLC6A9	Q59GK1	Q6A0F2
ATP8B1	OTTHUMP00000169147	Q71RC8	SLC7A3	Q59GP0	Q6A0F3
ATP8B2	OTTHUMP00000169536	Q75N13	SLC8A1	Q5JPP8	Q6AI14
ATP8B3	OTTHUMP00000169540	Q75RY0	SLC8A2	Q5K3P7	Q6ICJ5
ATP8B4	OTTHUMP00000170920	Q75T53	SLC8A3	Q5M8T2	Q6LAJ8
ATP9A	OTTHUMP00000172223	Q762B6	SLC9A1	Q5PT55	Q6LAJ9
ATP9B	OTTHUMP00000172224	Q76EJ3	SLC9A10	Q5SXD5	Q6MZW7
B0AZS0	OTTHUMP00000172594	Q7KZM7	SLC9A11	Q5SXE0	Q6P4C1
B0FYA3	OTTHUMP00000172778	Q7L273	SLC9A2	Q5T8R5	Q6PEL1
B0QYB1	OTTHUMP00000172786	Q7LBE3	SLC9A3	Q5TAB8	Q6PXP3
B0QYB2	OTTHUMP00000173071	Q7RTX7	SLC9A4	Q5TAH2	Q6U841

B0S7E9	OTTHUMP00000173073	Q7Z2E7	SLC9A5	Q5TBB2	Q6UWJ1
B0S8I9	OTTHUMP00000173074	Q7Z2H8	SLC9A6	Q5TBB3	Q6YIQ9
B1AKY9	OTTHUMP00000173075	Q7Z2W7	SLC9A7	Q5TF39	Q6ZMM8
B1AKZ1	OTTHUMP00000173076	Q7Z2W7-2	SLC9A8	Q5TIE1	Q6ZUB2
B1AL64	OTTHUMP00000173720	Q7Z2W7-3	SLC9A9	Q5U5S6	Q76EJ3
B1ALJ8	OTTHUMP00000173721	Q7Z3J4	SLCO1A2	Q5VVV3	Q7LBE3
B1ALM3	OTTHUMP00000173723	Q7Z3S7	SLCO1B1	Q5VVV4	Q7Z2E7
B1AMF4	OTTHUMP00000173724	Q7Z418	SLCO1B3	Q5VVV5	Q7Z2H8
B1AMV8	OTTHUMP00000173853	Q7Z442	SLCO2B1	Q5VVV6	Q7Z4G5
B1AQK4	OTTHUMP00000173854	Q7Z443	STX1B	Q5VVV7	Q7Z5R3
B1AQK6	OTTHUMP00000174382	Q7Z486	SVOP	Q5VVV9	Q7Z7F3
B1AQK7	OTTHUMP00000174457	Q7Z4D6	TCIRG1	Q5VVW0	Q7Z7F4
B1AVM8	OTTHUMP00000174643	Q7Z4G5	TCN1	Q5VVW5	Q86UD5
B1AW85	OTTHUMP00000175493	Q7Z4N2	TCN2	Q5W1L7	Q86VS0
B1N7F7	OTTHUMP00000175495	Q7Z4Y8	TMCO3	Q5W1L8	Q86WA9
B1N7F8	OTTHUMP00000176030	Q7Z5R3	TMEM37	Q5Y190	Q8IV21
B1N7G2	OTTHUMP00000176253	Q7Z5Y7	TMEM38A	Q659E4	Q8IVB4
B1N7G4	OTTHUMP00000176523	Q7Z678	TMEM38B	Q695T7	Q8IXG2
B1N7G7	OTTHUMP00000177496	Q7Z7F3	TNFAIP1	Q6A0F2	Q8IYC9
B2RAZ0	OTTHUMP00000177671	Q7Z7F4	TNNI2	Q6A0F3	Q8IYV4
B2RTU0	OTTHUMP00000178112	Q86UD5	TOMM40	Q6AI14	Q8N130
B2RUT3	OTTHUMP00000178113	Q86VF9	TOMM40L	Q6ICJ5	Q8N753
B3KNX4	OTTHUMP00000178471	Q86VS0	TPCN1	Q6J4K2	Q8NBD6
B3KP57	OTTHUMP00000181626	Q86W47	TPCN2	Q6LAJ8	Q8NBS3
B3KPN6	OTTHUMP00000181628	Q86WA9	TRAPPC10	Q6LAJ9	Q8NCC2

B3KQH9	OTTHUMP00000181864	Q86XG6	TRPA1	Q6MZW7	Q8NCC5
B3KQQ6	OTTHUMP00000183027	Q86XI1	TRPC1	Q6P4C1	Q8TBM6
B3KS22	OTTHUMP00000183035	Q86XN5	TRPC3	Q6PEL1	Q8TCC2
B3KSD7	OTTHUMP00000196015	Q86XQ3	TRPC4	Q6PXP3	Q8TCC7
B3KSD9	OTTHUMP00000196116	Q86YM0	TRPC5	Q6U841	Q8TD20
B3KSG7	OTTHUMP00000196119	Q8IU99	TRPC6	Q6UWJ1	Q8TDB8
B3KSI8	OTTHUMP00000196122	Q8IUZ4	TRPC7	Q6YBV0	Q8TE54
B3KSM4	OTTHUMP00000196213	Q8IV15	TRPM1	Q6YIQ9	Q8TED4
B3KSN7	OTTHUMP00000196734	Q8IV21	TRPM2	Q6ZMM8	Q8WUM9
B3KSZ6	OTTHUMP00000196738	Q8IV77	TRPM3	Q6ZSM3	Q8WW56
B3KTR3	OTTHUMP00000196742	Q8IVB4	TRPM4	Q6ZUB2	Q8WWT9
B3KTU1	OTTHUMP00000196921	Q8IVJ1	TRPM5	Q71RS6	Q8WWX8
B3KTU2	OTTHUMP00000196923	Q8IVZ4	TRPM6	Q76EJ3	Q92581
B3KU47	OTTHUMP00000196997	Q8IW23	TRPM7	Q7LBE3	Q92911
B3KU87	OTTHUMP00000198656	Q8IW96	TRPM8	Q7RTX9	Q969S0
B3KUF1	OTTHUMP00000198657	Q8IWP7	TRPV1	Q7RTY0	Q96A29
B3KVU0	OTTHUMP00000198782	Q8IWT1	TRPV2	Q7RTY1	Q96AA3
B3KW93	OTTHUMP00000198784	Q8IWU4	TRPV3	Q7Z2E7	Q96BK0
B3KXZ3	OTTHUMP00000198785	Q8IXG2	TRPV4	Q7Z2H8	Q96CD3
B3KY17	OTTHUMP00000198950	Q8IXW4	TRPV5	Q7Z4G5	Q96EP9
B3SZS3	OTTHUMP00000198951	Q8IYC9	TRPV6	Q7Z5R3	Q96G79
BEST1	OTTHUMP00000199574	Q8IYR7	TTYH1	Q7Z7F3	Q96KH8
BEST2	OTTHUMP00000199749	Q8IYV4	TTYH2	Q7Z7F4	Q96MZ1
BEST3	OTTHUMP00000200487	Q8IYY2	TTYH3	Q86UD5	Q96N87
BEST4	OTTHUMP00000200880	Q8IZC9	UNQ5929	Q86VL8	Q96Q91

BSND	OTTHUMP00000200882	Q8IZF0	UQCR	Q86VS0	Q96QG1
BTBD10	OTTHUMP00000201041	Q8IZK6	UQCR10	Q86WA9	Q96QG2
C15orf27	OTTHUMP00000201042	Q8IZS8	UQCRB	Q86YT5	Q96RN1
CACNA1A	OTTHUMP00000201043	Q8N130	UQCRC1	Q8IV21	Q96T83
CACNA1B	OTTHUMP00000201044	Q8N1C3	UQCRFS1	Q8IVB4	Q99884
CACNA1C	OTTHUMP00000201045	Q8N1M1	UQCRFSL1	Q8IXG2	Q9BS91
CACNA1D	OTTHUMP00000201046	Q8N1M1-1	UQCRH	Q8IY34	Q9BSB7
CACNA1E	OTTHUMP00000201179	Q8N1Q4	UQCRQ	Q8IYC9	Q9BSL2
CACNA1F	P00156	Q8N1S5	VDAC1	Q8IYV4	Q9BW45
CACNA1G	P00450	Q8N3X5	VDAC2	Q8N130	Q9BW84
CACNA1H	P00846	Q8N4V2	VDAC3	Q8N695	Q9BXS9
CACNA1I	P02708	Q8N5I3	XP_001126945	Q8N697	Q9BY07
CACNA1S	P02708-2	Q8N5Z5	XP_001127670	Q8N753	Q9BYW1
CACNA2D1	P02730	Q8N753	XP_001128895	Q8NBD6	Q9BYZ7
CACNA2D2	P03928	Q8N880	XP_001129849	Q8NBS3	Q9GZN6
CACNA2D3	P04839	Q8N8W8	XP_001131246	Q8NCC2	Q9GZV3
CACNA2D4	P04920	Q8N8Y2	XP_001132790	Q8NCC5	Q9H015
CACNB1	P05023	Q8NB49	XP_001714015	Q8NCK7	Q9H021
CACNB2	P05026	Q8NBD6	XP_001714183	Q8NDX2	Q9H1V8
CACNB3	P05496	Q8NBS3	XP_001714554	Q8NFF2	Q9H2B4
CACNB4	P06576	Q8NC69	XP_001714623	Q8NG04	Q9H2H9
CACNG1	P07510	Q8NCC2	XP_001714645	Q8TBM6	Q9H2J7
CACNG2	P07919	Q8NCC5	XP_001714789	Q8TCC2	Q9H2X9
CACNG3	P08195	Q8NCM2	XP_001716524	Q8TCC7	Q9H598
CACNG4	P09131	Q8NEC5	XP_001716897	Q8TD20	Q9HAS3

CACNG5	P0C7P4	Q8NER1	XP_001717927	Q8TDB8	Q9HC58
CACNG6	P11166	Q8NET8	XP_001718305	Q8TE54	Q9HCJ1
CACNG7	P11168	Q8NET8-3	XP_001718387	Q8TED4	Q9NP91
CACNG8	P11169	Q8NEW0	XP_001718688	Q8WUM9	Q9NQU1
CATSPER1	P11230	Q8NEY4	XP_001718903	Q8WV87	Q9NRA2
CATSPER2	P13569	Q8NFU0	XP_001719419	Q8WW56	Q9NRM0
CATSPER3	P13637	Q8NFU1	XP_001720043	Q8WWT9	Q9NSA0
CATSPER4	P13866	Q8NHE4	XP_001720109	Q8WWX8	Q9NSD5
CAVB2F	P14415	Q8NHX9	XP_001720113	Q92536	Q9NTN3
CCS	P14672	Q8TAD4	XP_001721108	Q92581	Q9NW50
CFTR	P14867	Q8TAE7	XP_001721319	Q92911	Q9NY64
CHRNA1	P14927	Q8TBC3	XP_001722085	Q969I6	Q9NY91
CHRNA10	P15313	Q8TBM6	XP_001722388	Q969S0	Q9P2U7
CHRNA2	P15382	Q8TCC2	XP_001722727	Q96A29	Q9UBY0
CHRNA3	P16389	Q8TCC7	XP_001726222	Q96AA3	Q9UDJ1
CHRNA4	P16615	Q8TCU5	XP_001726980	Q96BI1	Q9UGH3
CHRNA5	P17302	Q8TD20	XP_932946	Q96BK0	Q9UGQ3
CHRNA6	P17658	Q8TD22	XP_947421	Q96CD3	Q9UHW9
CHRNA7	P17787	Q8TD43	ZACN	Q96EP9	Q9UKG4
CHRNA7-2	P18505	Q8TD43-2	ZIC2	Q96FL8	Q9UN76
CHRNA9	P18507	Q8TDB8	hCG_2015407	Q96G79	Q9UP95
CHRNB1	P18859	Q8TDD5	hCG_2015786	Q96KH8	Q9UPR5
CHRNB2	P19634	Q8TDN1	kcnj12x	Q96MZ1	Q9UQ04
CHRNB3	P20020	Q8TDN2	ncx1	Q96N87	Q9Y289
CHRNB4	P20061	Q8TE54	KCNS3	Q96Q91	Q9Y2C5

CHRND	P20062	Q8TED4	KCNT1	Q96QD8	Q9Y2D2
CHRNE	P20648	Q8TEG5	KCNT2	Q96QG1	Q9Y2W3
CHRNG	P21281	Q8TEH0	KCNU1	Q96QG2	Q9Y345
CLCA1	P21283	Q8TF62	KCNV1	Q96RN1	Q9Y666
CLCA2	P21796	Q8WTS0	KCNV2	Q96T83	Q9Y694
CLCA3	P21817	Q8WUM9	KCTD1	Q99624	Q9Y6L6
CLCN1	P22001	Q8WVF5	KCTD10	Q99884	Q9Y6M7
CLCN2	P22459	Q8WW53	KCTD11	Q9BRI2	Q9Y6R1
CLCN3	P22460	Q8WW56	KCTD12	Q9BS91	Q9Y6R3
CLCN4	P22732	Q8WWG8	KCTD13	Q9BSB7	RESDA1
CLCN5	P23415	Q8WWG9	KCTD14	Q9BSL2	RFT1
CLCN6	P23416	Q8WWN6	KCTD15	Q9BTX8	SCL8A3
CLCN7	P23634	Q8WWT9	KCTD16	Q9BW45	SLC10A1
CLCNKA	P24046	Q8WWX8	KCTD17	Q9BW84	SLC10A2
CLCNKB	P24539	Q8WXA8	KCTD18	Q9BXS9	SLC10A3
CLDN16	P25705	Q8WXG7	KCTD19	Q9BY07	SLC10A4
CLIC1	P27352	Q8WXI7	KCTD2	Q9BY10	SLC10A5
CLIC2	P27449	Q8WXS4	KCTD20	Q9BYW1	SLC10A6
CLIC3	P28472	Q8WXS5	KCTD21	Q9BYZ7	SLC10A7
CLIC4	P28476	Q8WY07	KCTD3	Q9BZV2	SLC12A1
CLIC5	P29973	Q8WZ19	KCTD4	Q9BZW2	SLC12A2
CLIC6	P2RX1	Q8WZ55	KCTD5	Q9GZN6	SLC12A3
CNGA1	P2RX2	Q92504	KCTD6	Q9GZV3	SLC12A4
CNGA2	P2RX3	Q92581	KCTD7	Q9H015	SLC12A5
CNGA3	P2RX4	Q92736	KCTD8	Q9H021	SLC12A6

CNGA4	P2RX5	Q92806	KCTD9	Q9H1K4	SLC12A7
CNGB1	P2RX6	Q92887	KCTD9L	Q9H1V8	SLC13A2
CNGB3	P2RX7	Q92911	KIAA1282	Q9H2B4	SLC13A3
CP	P30049	Q92952	LASP1	Q9H2H9	SLC13A4
CSPG4	P30531	Q92953	LOC202789	Q9H2J7	SLC15A1
CUL5	P30532	Q93034	LRCH1	Q9H2X9	SLC15A2
CYBB	P30926	Q93050	MCOLN1	Q9H598	SLC17A5
DKFZp434P0831	P31639	Q93084	MCOLN2	Q9H936	SLC17A7
DKFZp566I1346	P31644	Q93086	MCOLN3	Q9HAS3	SLC1A1
DKFZp686H2042	P31749	Q969M1	MLC1	Q9HC58	SLC1A2
DKFZp686H2093	P31930	Q969S0	MON2	Q9HCJ1	SLC1A3
DKFZp686I0955	P32297	Q96A05	MORC3	Q9NP91	SLC1A4
DKFZp686P10213	P32418	Q96A29	MRS2	Q9NQU1	SLC1A5
DKFZp761D171	P34903	Q96AA3	MS4A2	Q9NRA2	SLC1A6
DKFZp779H1459	P35498	Q96BK0	MST103	Q9NRM0	SLC1A7
DKFZp781L0846	P35499	Q96CD3	MT-ATP6	Q9NSA0	SLC20A1
EN1	P35523	Q96CX2	MT-ATP8	Q9NSD5	SLC20A2
ENSP00000216568	P35670	Q96D31	MT-CYB	Q9NTN3	SLC22A4
ENSP00000244759	P35670-4	Q96D96	MUC16	Q9NW50	SLC22A5
ENSP00000254589	P36542	Q96DB9	NAGLT1	Q9NY64	SLC22A6
ENSP00000258538	P36543	Q96EP9	NALCN	Q9NY91	SLC22A7
ENSP00000317952	P36544	Q96FT7	NBC	Q9P2U7	SLC22A8
ENSP00000319241	P37088	Q96G79	NHEDC1	Q9P2U8	SLC23A2
ENSP00000322354	P37088-2	Q96GZ6	Q16281	Q9UBX3	SLC24A1
ENSP00000331410	P37088-4	Q96H72	Q16322	Q9UBY0	SLC24A3

ENSP00000342896	P38606	Q96JW4	Q16348	Q9UDJ1	SLC25A11
ENSP00000347944	P39086	Q96KH7	Q16445	Q9UGH3	SLC25A3
ENSP00000350560	P39086-2	Q96KH8	Q16478	Q9UGQ3	SLC26A1
ENSP00000351597	P40879	Q96KK3	Q16515	Q9UHI7	SLC26A11
ENSP00000359129	P42261	Q96L42	Q16558	Q9UHW9	SLC26A2
ENSP00000363337	P42262	Q96LB4	Q16595	Q9UI40	SLC26A3
ENSP00000364933	P42262-2	Q96MP8	Q16720	Q9UKG4	SLC26A4
ENSP00000366546	P42263	Q96MZ1	Q16864	Q9UN76	SLC26A5
ENSP00000370296	P43003	Q96N87	Q17R43	Q9UP95	SLC26A6
ENSP00000370336	P43004	Q96NB2	Q17RG1	Q9UPR5	SLC26A7
ENSP00000371078	P43005	Q96NY7	Q17RM9	Q9UPY5	SLC26A8
ENSP00000371255	P43007	Q96P03	Q17RT3	Q9UQ04	SLC26A9
ENSP00000371447	P43681	Q96P05	Q17RZ6	Q9Y289	SLC28A1
ENSP00000374097	P45880	Q96P56	Q19QZ7	Q9Y2C5	SLC28A2
ENSP00000374283	P46059	Q96PH1	Q1WWK6	Q9Y2D2	SLC28A3
ENSP00000376488	P46098	Q96PR1	Q20BI4	Q9Y2W3	SLC2A1
ENSP00000377481	P46721	Q96Q91	Q29RF8	Q9Y345	SLC2A10
ENSP00000378213	P47869	Q96QG1	Q2M1U9	Q9Y666	SLC2A11
ENSP00000380229	P47870	Q96QG2	Q2M2N9	Q9Y694	SLC2A12
ENSP00000380895	P47985	Q96QT4	Q2M3C6	Q9Y6L6	SLC2A14
ENSP00000384453	P48047	Q96RN1	Q2TB63	Q9Y6M7	SLC2A2
ENSP00000385155	P48048	Q96RP8	Q2VI00	Q9Y6R1	SLC2A3
ENSP00000385389	P48048-2	Q96RY8	Q2Y0W8	Q9Y6R3	SLC2A4
ENSP00000385395	P48050	Q96SC1	Q2YD85	RESDA1	SLC2A5
EPB3	P48051	Q96SI1	Q32MB6	RFT1	SLC2A6

Em:AP000350.2	P48058	Q96T54	Q36731	SCL8A3	SLC2A7
FAM26C	P48065	Q96T55	Q3KNS8	SLC10A1	SLC2A8
FETA	P48066	Q96T83	Q3KNW5	SLC10A2	SLC32A1
FKBP12.6	P48067	Q99250	Q3LIB2	SLC10A3	SLC34A1
FKBP1A	P48167	Q99250-2	Q3LU46	SLC10A4	SLC34A2
FLJ00227	P48169	Q99415	Q3LU47	SLC10A5	SLC34A3
FLJ00232	P48201	Q99437	Q3MIQ9	SLC10A6	SLC35A1
FXN	P48544	Q99500	Q3YAB7	SLC10A7	SLC35A2
FXYD1	P48547	Q99571	Q3YAB9	SLC12A1	SLC35A3
FXYD2	P48549	Q99572	Q3YAC3	SLC12A2	SLC35A4
FXYD3	P48553	Q99712	Q3YAC5	SLC12A3	SLC35A5
FXYD4	P48664	Q99726	Q3YAC9	SLC12A4	SLC35B1
FXYD5	P48751	Q99766	Q3YAD0	SLC12A5	SLC35B4
FXYD6	P48764	Q99884	Q3YAD3	SLC12A6	SLC35C1
FXYD7	P48788	Q99928	Q401N2	SLC12A7	SLC35D1
FXYD8	P48995	Q9B185	Q495K2	SLC12A8	SLC35D2
GABRA1	P49281	Q9BQ13	Q495M3	SLC13A1	SLC35D3
GABRA2	P49790	Q9BQ31	Q495M3-2	SLC13A2	SLC36A1
GABRA3	P50443	Q9BQS7	Q495M3-3	SLC13A3	SLC36A2
GABRA4	P50993	Q9BRI3	Q496J0	SLC13A4	SLC37A1
GABRA5	P51164	Q9BRR4	Q496J2	SLC13A5	SLC37A2
GABRA6	P51168	Q9BRY0	Q49AB7	SLC15A1	SLC37A3
GABRB1	P51168-2	Q9BS91	Q9Y226	SLC15A2	SLC37A4
GABRB2	P51170	Q9BSA4	Q9Y257	SLC15A3	SLC38A1
GABRB3	P51172	Q9BSB7	Q9Y267	SLC15A4	SLC3A2

GABRD	P51575	Q9BSF8	Q9Y277	SLC16A1	SLC45A1
GABRE	P51787	Q9BSL2	Q9Y289	SLC16A11	SLC4A1
GABRG1	P51788	Q9BW45	Q9Y2C5	SLC16A12	SLC4A10
GABRG2	P51790	Q9BW84	Q9Y2D2	SLC16A13	SLC4A11
GABRG3	P51793	Q9BW91	Q9Y2G3	SLC16A14	SLC4A2
GABRP	P51795	Q9BWM7	Q9Y2Q0	SLC16A2	SLC4A3
GABRQ	P51797	Q9BX84	Q9Y2U2	SLC16A3	SLC4A4
GABRR1	P51798	Q9BXS9	Q9Y2W3	SLC16A4	SLC4A5
GABRR2	P51800	Q9BXT2	Q9Y2W7	SLC16A5	SLC4A7
GABRR3	P51801	Q9BY07	Q9Y342	SLC16A6	SLC4A8
GIF	P53794	Q9BY48	Q9Y345	SLC16A7	SLC5A1
GJA1	P54284	Q9BYW1	Q9Y3Q4	SLC16A8	SLC5A11
GLRA1	P54289	Q9BYZ7	Q9Y487	SLC17A5	SLC5A2
GLRA2	P54707	Q9C0H2	Q9Y597	SLC17A6	SLC5A3
GLRA3	P54709	Q9C0K1	Q9Y5I7	SLC17A7	SLC5A4
GLRA4	P54710	Q9GZN6	Q9Y5K8	SLC17A8	SLC5A5
GLRB	P55011	Q9GZQ4	Q9Y5S1	SLC19A1	SLC5A7
GLUR5	P55017	Q9GZU1	Q9Y5S8	SLC19A2	SLC6A1
GRIA1	P56134	Q9GZV3	Q9Y5Y9	SLC19A3	SLC6A11
GRIA2	P56373	Q9GZZ6	Q9Y666	SLC1A1	SLC6A12
GRIA3	P56381	Q9H015	Q9Y691	SLC1A2	SLC6A13
GRIA4	P56385	Q9H021	Q9Y694	SLC1A3	SLC6A14
GRID1	P56696	Q9H0N0	Q9Y694-4	SLC1A4	SLC6A15
GRID2	P57057	Q9H0Q3	Q9Y696	SLC1A5	SLC6A16
GRIK1	P57103	Q9H1D0	Q9Y698	SLC1A6	SLC6A17

GRIK2	P57789	Q9H1D0-2	Q9Y6H6	SLC1A7	SLC6A18
GRIK3	P58549	Q9H1V8	Q9Y6J6	SLC20A1	SLC6A19
GRIK4	P58550	Q9H252	Q9Y6L6	SLC20A2	SLC6A2
GRIK5	P58743	Q9H2B4	Q9Y6M5	SLC22A18	SLC6A20
GRIN1	P59646	Q9H2H9	Q9Y6M7	SLC22A4	SLC6A5
GRIN2A	P61266	Q9H2J7	Q9Y6N3	SLC22A5	SLC6A7
GRIN2B	P61421	Q9H2S1	Q9Y6R1	SLC22A6	SLC6A9
GRIN2C	P62942	Q9H2X9	Q9Y6R3	SLC22A7	SLC8A1
GRIN2D	P62955	Q9H310	RAB6C	SLC22A8	SLC8A2
GRIN3A	P63252	Q9H313	RCNC2	SLC23A1	SLC8A3
GRIN3B	P68106	Q9H3F6	RESDA1	SLC23A2	SLC9A1
GRM7	P78334	Q9H3M0	RFT1	SLC24A1	SLC9A10
HCN1	P78348	Q9H427	RHAG	SLC24A2	SLC9A11
HCN2	P78381	Q9H598	RHBG	SLC24A3	SLC9A2
HCN3	P78382	Q9H6F2	RHCG	SLC24A4	SLC9A3
HCN4	P78383	Q9H7F0	RP11-141J10.3-009	SLC24A5	SLC9A4
HEPH	P78488	Q9H9B4	RP11-257K9.5-005	SLC24A6	SLC9A5
HEPHL1	P78508	Q9HAS3	RP11-30G24.1-003	SLC25A10	SLC9A6
HIT000005340	P98194	Q9HAT3	RP11-5F19.1-003	SLC25A11	SLC9A7
HIT000019828	P98194-1	Q9HAT6	RP5-955M13.1-001	SLC25A16	SLC9A8
HIT000025397	P98194-2	Q9HB14	RYR1	SLC25A18	SLC9A9
HIT000047836	P98194-5	Q9HB15		SLC25A22	SLCO1B1
HIT000070322	P98194-6	Q9HBA0		SLC25A3	SLCO2B1
HIT000088536	P98196	Q9HBG1		SLC25A6	TMCO3
HIT000191698	P98198	Q9HBG4		SLC26A1	XP_001720043

HIT000195934	PEA15	Q9HBH2	SLC26A10	XP_001721108
HIT000217122	PKD1L2	Q9HC58	SLC26A11	XP_001722727
HIT000262994	PKD1L3	Q9HCF6	SLC26A2	
HIT000296330	PKD2L1	Q9HCJ1	SLC26A3	
HIT000303363	PKD2L2	Q9HCX4	SLC26A4	
HIT000321000	PKDREJ	Q9HD20	SLC26A5	
HTR3A	PLLP	Q9HD23	SLC26A6	
HTR3B	PLP2	Q9NP59	SLC26A7	
HTR3C	PMCA1	Q9NP91	SLC26A8	
HTR3D	PRES	Q9NP94	SLC26A9	
HTR3E	Q00325	Q9NPA1	SLC28A1	
HVCN1	Q00975	Q9NPC2	SLC28A2	
ITPR1	Q01118	Q9NPD5	SLC28A3	
ITPR2	Q01362	Q9NPI9	SLC2A1	
ITPR3	Q01668	Q9NQ11	SLC2A10	
KCNA1	Q01814	Q9NQA5	SLC2A11	
KCNA10	Q01814-1	Q9NQU1	SLC2A12	
KCNA2	Q01814-5	Q9NQW8	SLC2A14	
KCNA3	Q01814-6	Q9NQW8-2	SLC2A2	
KCNA4	Q02094	Q9NR82	SLC2A3	
KCNA5	Q02641	Q9NRA2	SLC2A4	
KCNA6	Q02978	Q9NRM0	SLC2A5	
KCNA7	Q03721	Q9NS40	SLC2A6	
KCNAB1	Q04656	Q9NS61	SLC2A7	
KCNAB2	Q04671	Q9NSA0	SLC2A8	

KCNAB3	Q04844	Q9NSA2	SLC32A1
KCNB1	Q04941	Q9NSD5	SLC34A1
KCNB2	Q05586	Q9NTG1	SLC34A2
KCNC1	Q05901	Q9NTI2	SLC34A3
KCNC2	Q05925	Q9NTN3	SLC35A1
KCNC3	Q05BQ3	Q9NUM3	SLC35A2
KCNC4	Q05CS9	Q9NVV0	SLC35A3
KCND1	Q06055	Q9NW50	SLC35A4
KCND2	Q06432	Q9NW63	SLC35A5
KCND3	Q06495	Q9NWI4	SLC35B1
KCNE1	Q07001	Q9NX22	SLC35B4
KCNE2	Q07699	Q9NXV2	SLC35C1
KCNE3	Q08289	Q9NY12	SLC35D1
KCNE4	Q08357	Q9NY26	SLC35D2
KCNF1	Q08AQ2	Q9NY37	SLC35D3
KCNG1	Q09428	Q9NY46	SLC36A1
KCNG2	Q09470	Q9NY47	SLC36A2
KCNG3	Q0GE19	Q9NY64	SLC36A4
KCNG4	Q0VAK9	Q9NY72	SLC37A1
KCNH1	Q12791	Q9NY91	SLC37A2
KCNH2	Q12791-4	Q9NYG8	SLC37A3
KCNH4	Q12791-7	Q9NYZ2	SLC37A4
KCNH5	Q12809	Q9NYZ2-2	SLC38A1
KCNH6	Q12835	Q9NYZ2-4	SLC38A2
KCNH7	Q12879	Q9NZA1	SLC38A3

KCNH8	Q12908	Q9NZI2	SLC38A4
KCNIP1	Q13002	Q9NZM6	SLC3A2
KCNIP2	Q13003	Q9NZQ8	SLC45A1
KCNIP3	Q13032	Q9NZV8	SLC46A2
KCNIP4	Q13183	Q9P012	SLC47A1
KCNJ1	Q13224	Q9P0L9	SLC47A2
KCNJ10	Q13303	Q9P0X4	SLC4A1
KCNJ11	Q13423	Q9P1Z3	SLC4A10
KCNJ12	Q13433	Q9P241	SLC4A11
KCNJ13	Q13488	Q9P2U7	SLC4A2
KCNJ14	Q13507	Q9UBD6	SLC4A3
KCNJ15	Q13563	Q9UBL9	SLC4A4
KCNJ16	Q13621	Q9UBL9-5	SLC4A5
KCNJ2	Q13698	Q9UBN1	SLC4A7
KCNJ3	Q13717	Q9UBN4	SLC4A8
KCNJ4	Q13733	Q9UBY0	SLC5A1
KCNJ5	Q13796	Q9UD79	SLC5A11
KCNJ6	Q13829	Q9UD80	SLC5A12
KCNJ8	Q13936	Q9UDJ1	SLC5A2
KCNJ9	Q14003	Q9UDW1	SLC5A3
KCNK1	Q14028	Q9UF02	SLC5A4
KCNK10	Q14500	Q9UGH3	SLC5A5
KCNK12	Q14524	Q9UGM1	SLC5A7
KCNK13	Q14571	Q9UGQ3	SLC5A8
KCNK15	Q14573	Q9UHB0	SLC6A1

KCNK16	Q14643	Q9UHC3	SLC6A11
KCNK17	Q14654	Q9UHW9	SLC6A12
KCNK18	Q14681	Q9UI12	SLC6A13
KCNK2	Q14721	Q9UI33	SLC6A14
KCNK3	Q14722	Q9UIX4	SLC6A15
KCNK4	Q14802	Q9UJ96	SLC6A16
KCNK5	Q14831	Q9UK17	SLC6A17
KCNK6	Q14847	Q9UKG4	SLC6A18
KCNK7	Q14916	Q9UL51	SLC6A19
KCNK9	Q14940	Q9UL62	SLC6A2
KCNMA1	Q14957	Q9ULD8	SLC6A20
KCNMB1	Q14973	Q9ULF5	SLC6A3
KCNMB2	Q14DR2	Q9ULK0	SLC6A4
KCNMB3	Q14DU4	Q9ULQ1	SLC6A5
KCNMB3L	Q15043	Q9ULS6	SLC6A6
KCNMB4	Q15049	Q9UN42	SLC6A7
KCNN1	Q15121	Q9UN76	SLC6A8
KCNN2	Q15413	Q9UN88	SLC6A9
KCNN3	Q15739	Q9UNN1	SLC7A11
KCNN4	Q15758	Q9UNX9	SLC7A6
KCNQ1	Q15822	Q9UP79	SLC8A1
KCNQ2	Q15825	Q9UP95	SLC8A2
KCNQ3	Q15842	Q9UPR5	SLC8A3
KCNQ4	Q15858	Q9UQ04	SLC9A1
KCNQ5	Q15878	Q9UQ05	SLC9A10

KCNRG	Q15904	Q9UQC9	SLC9A11	
KCNS1	Q16099	Q9UQD0	SLC9A2	
KCNS2	Q16280	Q9Y210	SLC9A3	

Table S3. Characteristics of participants in this study

Characteristics	Control	HUA
Number	2945	1387
Male (%)	70.53%	76.42%
Age	69.00 (9.25)	67.91 (11.93)
Height (cm)	161.37 (7.52)	162.63 (7.54)
Weight (kg)	63.71 (9.96)	68.56 (11.12)
BMI	24.43 (3.24)	25.88 (3.52)
Serum Urate (umol/l)	300.73 (63.13)	482.30 (61.14)
total bilirubin (umol/l)	19.39 (8.39)	18.98 (7.59)
Glucose (mmol/L)	5.60 (1.67)	5.59 (1.31)
Cholesterol (mmol/L)	4.75 (0.93)	5.06 (1.52)
Triglyceride (mmol/L)	1.51 (0.96)	2.23 (1.76)
Creatinine (umol/L)	71.75 (17.61)	89.80 (29.24)
blood urea nitrogen (mmol/L)	5.48 (2.13)	6.24 (1.95)

HUA, hyperuricemia. Data was showed as mean (SD).

Table S4. The number of participants in this study countered by gender and BMI

**Number**	**Non-gout**	**Gout**
Total	4332	582
**Gender**		
Male	3137	509
Female	1194	52
**BMI**		
Underweight	99	-
Normal weight	2024	-
Overweight	1825	-

Data are shown as the mean (SD). BMI: underweight: BMI < 18.5; normal weight: 18.50 ≤ BMI < 25; overweight: BMI ≥ 25. In the present study, only non-gout individuals were divided into subgroups according to body mass index (BMI) values.

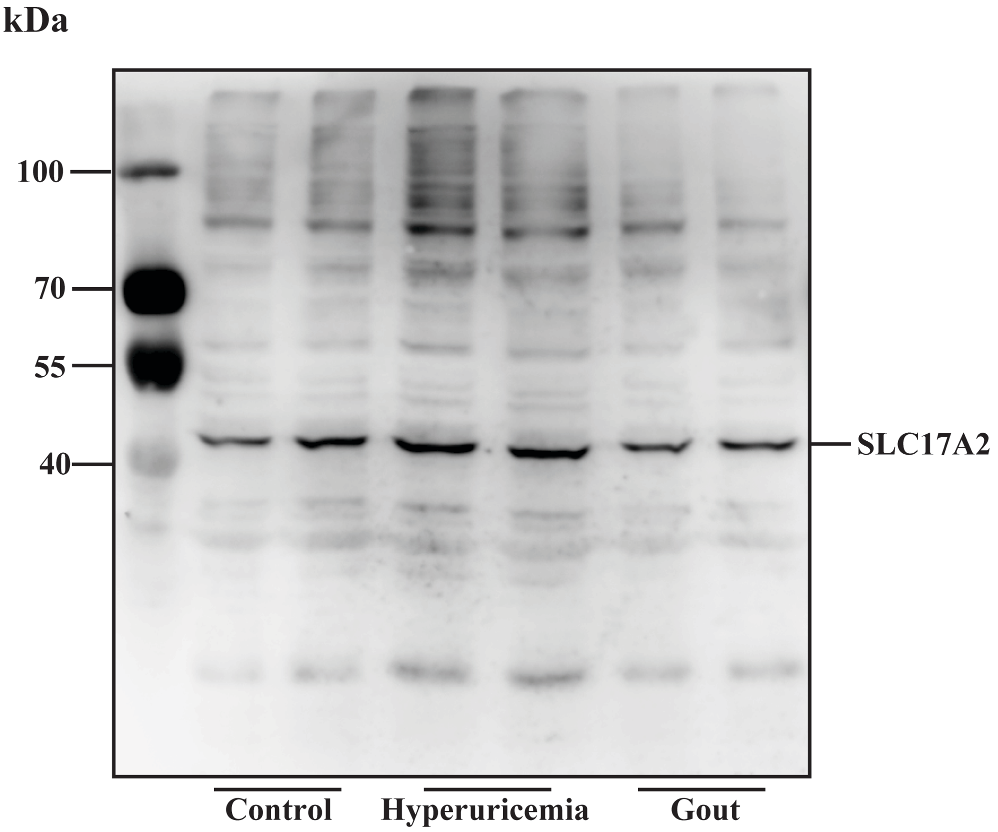

Figure S1. Full-length western blots analysis of SLC17A2 among groups

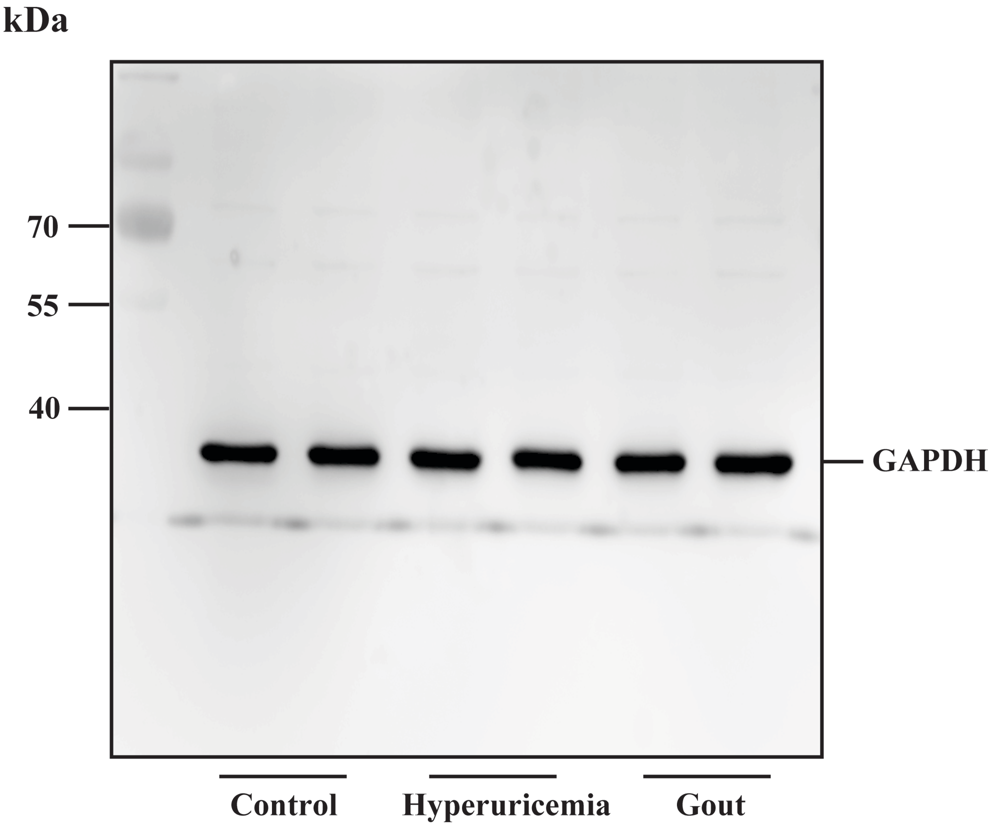

Figure S2. Full-length western blots analysis of GADPH among groups
